# Rapid changes in brain estrogen concentration during male sexual behavior are site and stimulus specific

**DOI:** 10.1038/s41598-021-99497-1

**Published:** 2021-10-11

**Authors:** Marie-Pierre de Bournonville, Catherine de Bournonville, Laura M. Vandries, Gwenaël Nys, Marianne Fillet, Gregory F. Ball, Jacques Balthazart, Charlotte A. Cornil

**Affiliations:** 1 GIGA Neurosciences, University of Liège, 15 Avenue Hippocrate, 4000 Liège, Belgium; 2 Center for Interdisciplinary Research on Medicines, University of Liège, Liège, Belgium; 3 Department of Psychology, University of Maryland, College Park, MD USA

**Keywords:** Motivation, Sexual behaviour, Animal behaviour

## Abstract

Classically, estrogens regulate male sexual behavior through effects initiated in the nucleus. However, neuroestrogens, i.e., estrogens locally produced in the brain, can act within minutes via membrane-initiated events. In male quail, rapid changes in brain aromatase activity occur after exposure to sexual stimuli. We report here that local extracellular estrogen concentrations measured by in vivo microdialysis increase during sexual interactions in a brain site- and stimulus-specific manner. Indeed, estrogen concentrations rose within 10 min of the initiation of sexual interaction with a female in the medial preoptic nucleus only, while visual access to a female led to an increase in estrogen concentrations only in the bed nucleus of the stria terminalis. These are the fastest fluctuations in local estrogen concentrations ever observed in the vertebrate brain. Their site and stimulus specificity strongly confirm the neuromodulatory function of neuroestrogens on behavior.

## Introduction

Steroids exert pervasive effects on brain, physiology and various behaviors ranging from learning and memory to socio-sexual behaviors^1,2^. This has been known for over a century and the identification of steroid binding sites in the brain was instrumental in dissecting the functional circuits that control these behaviors^3,4^. The molecular mechanisms mediating these effects remain however partly unknown. For example, if male sexual behavior is clearly activated by diffusible factors produced by the testes, as already established by Berthold in 1849^5^, the changes in brain concentrations of the steroids involved, testosterone and its metabolites, remain poorly characterized.

Estrogens, such as 17β-estradiol (E_2_), derived from testosterone aromatization in the brain mediate many central effects of testosterone^6^ and changes in brain aromatase activity (AA) have been described in a variety of physiological conditions and species^7–9^. The resulting changes in local E_2_ concentrations are less clear. A host of studies indicate that brain steroid concentrations are not a direct reflection of concentrations circulating in the blood (In rodents^10–12^; in quail^13^) which led to the discovery that the brain itself can synthesize steroids from cholesterol, independent from the supply of systemic precursors, these are the so-called neurosteroids^14,15^.

In quail, E_2_ activates aspects of male sexual behavior by acting both slowly as a transcription factor controlling the synthesis of proteins and more rapidly (within min) at the neuronal membrane in a mode similar to neurotransmitters or neuromodulators^16,17^. In parallel, the activity of brain aromatase, the enzyme producing estrogens^18–20^, is also regulated within two separate time frames: in the long term via changes in transcription and thus concentration of the enzyme^21^, and more rapidly (seconds to minutes) via phosphorylation of the enzyme triggered by changes in neuronal activity^22–24^. The view of a female or the sexual interaction with her affects within minutes brain AA specifically in nuclei that are part of the social behavior network^25^, such as the medial preoptic nucleus (POM) or bed nucleus of the stria terminalis (BST)^26,27^. However, these studies reflect AA measured *post-mortem* by an ex vivo assay designed to maximize enzymatic activity^28^ and do not directly answer the question of whether brain E_2_ concentrations also change in such a dynamic manner.

In vivo microdialysis has long been used to monitor dynamic changes in neurotransmitter concentrations in a behaviorally relevant context (e.g.,^29–31^), but until relatively recently it was thought that this approach could not be used for steroids, in particular E_2_ which is present in much lower concentrations. Remage-Healey and colleagues, however, showed with this technique that in zebra finches (*Taeniopygia guttata*), a species that displays an unusually high telencephalic AA, changes in E_2_ concentrations in the auditory cortex can be measured within 30 min after hearing conspecific songs^32^. We asked whether this approach would be sensitive enough to monitor changes in E_2_ concentrations in the POM of male quail, a brain region that exhibits a lower AA than the auditory cortex of zebra finches, though this activity is much higher than in the rodent brain^9,33^. By combining in vivo microdialysis with an ultra-sensitive ^125^I-based radioimmunoassay, we show here that brain estrogen concentrations can be monitored in the quail brain with a time resolution of 10 min. We demonstrate that these concentrations rapidly change during sexual interactions in a brain site- and stimulus-specific manner, which is consistent with the idea that these steroids are able to modulate behavior expression in a manner similar to a neuromodulator. Yet a causal relationship between these rapid changes in estrogens in a given region and behavior has not yet been demonstrated and further research will be necessary to firmly establish the existence of such a link.

## Results

We first characterized the dynamic properties of the microdialysis probes by incubating them in vitro in increasing or decreasing concentrations of E_2_ and measuring the steroid in the dialysate after various durations. This demonstrated that concentrations in the dialysate increase linearly with concentration outside the probe with a delay of 30 min and possibly less (See Supplementary information, SI2).

### Experiment 1: systemic in vivo injection of E_2_

**Figure 1:**
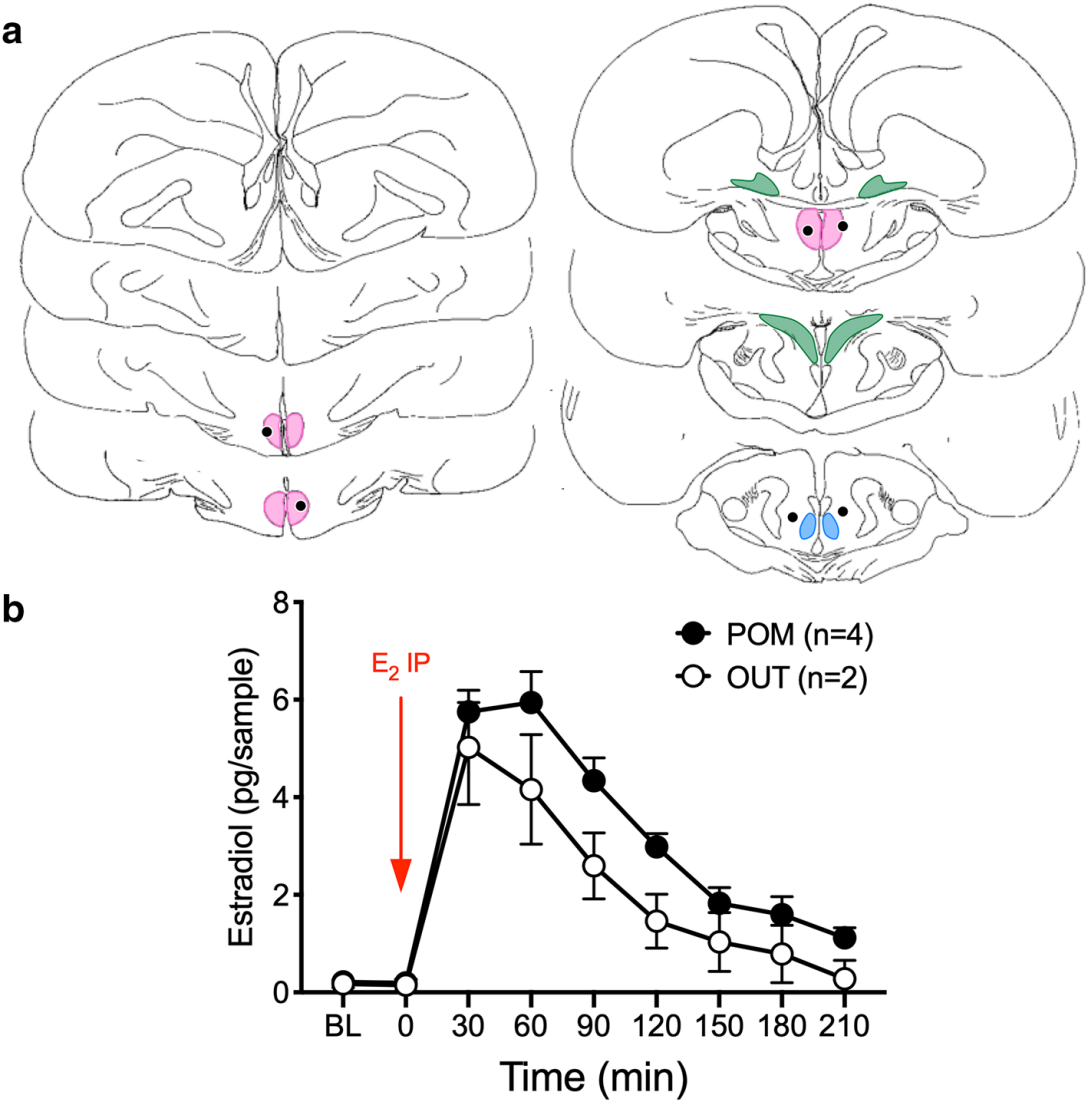
Effect of a peripheral injection of estradiol (E_2_) (500 µg/kg, i.p.) on the estrogen concentration in the dialysate as a function of the probe location. IN probes are either within or touching the colored zone representing the POM. (**a**) Schematic representation of coronal sections of the quail brain, from rostral (top left) to caudal (bottom right), showing the location of individual microdialysis probes (black dots). Colored shapes represent the distribution of aromatase positive cell populations as described in^65^: pink, Medial Preoptic Nucleus (POM); green, Bed nucleus of the Stria Terminalis (BST); blue, Ventromedial Nucleus of the hypothalamus (VMN). (**b**) Mean changes over time of estrogen concentration in the dialysate (mean ± S.D.). BL (baseline) represents the last point of a 60 min baseline period. The red arrow shows when E_2_ was injected intraperitoneally. A Friedman ANOVA detected significant changes over time in the POM group (χ^2^_n=4_ = 30.88, p = 0.0001). No statistical analysis was performed on the OUT group given that it contained only two individuals. Yet, these two individuals showed a similar pattern of response to E2 as those whose probe was in the POM, no obvious difference could be detected between the groups that both rapidly responded to the injection. Panel (**a**) is composed of home made drawings onto which probe positions were reported using Graphic for Mac (https://graphic.com/mac/), other panels were created with Graph Pad Prism 8 (https://www.graphpad.com/scientific-software/prism/). All panels were assembled in Graphic.

After a baseline of 60 min, males (n = 6) bearing microdialysis probes aimed at the POM were injected intraperitoneally with E_2_ (500 µg/kg, in propylene glycol: saline 4:1) and samples collected every 30 min. Estrogen concentration sharply increased in the dialysate of all birds within the first 30 min period after the injection of E_2_ independent of whether the probe was in POM (n = 4) or outside (OUT; n = 2) (Fig. 1). A gradual return to baseline followed over the next 3 h (see Fig. 1 for detail of statistical analyses).

### Experiment 2: testosterone retrodialysis

**Figure 2:**
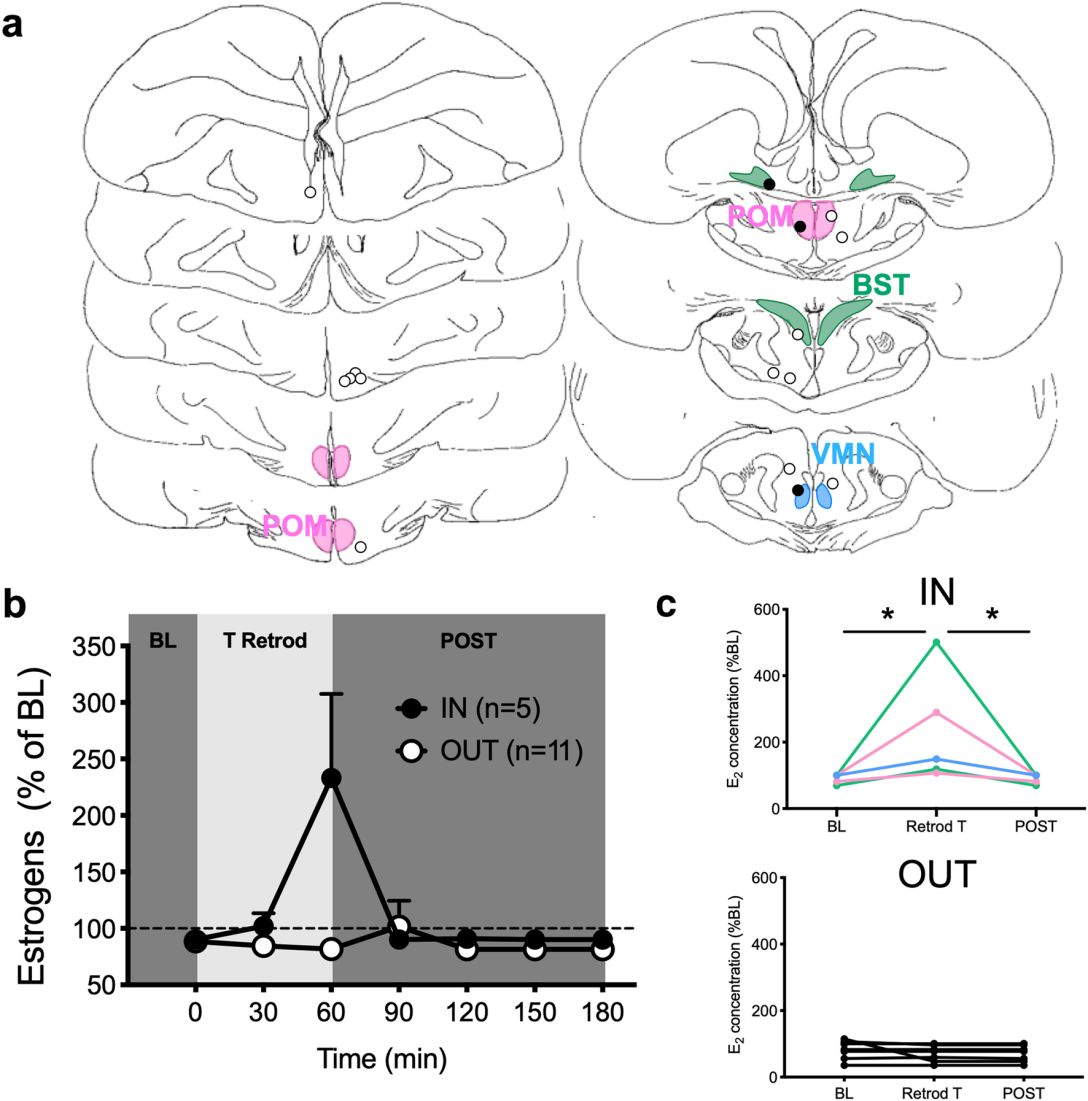
Effect of testosterone retrodialysis on the changes in estrogen concentrations in the dialysate expressed as a percentage of the baseline. (**a**) Schematic representation of coronal sections of the quail brain showing the location of the dialysis probes: IN probes are either within or touching the colored zones representing brain regions expressing aromatase. See legend of Fig. 1 for the definition of different brain areas. Black and open circles respectively represent birds in which estrogen concentrations did or did not increase above 150% of the BL during the retrodialysis of T. (**b**) Mean changes in time of estrogen concentration relative to the mean concentration measured in the 3 samples composing the baseline period. BL: baseline (last point of a 90 min baseline period), T Retrod: Testosterone retrodialysis, POST: Period after the end of testosterone retrodialysis. Only males with a probe in a brain region expressing aromatase exhibited a significant rise in estrogens peaking at the end of the retrodialysis period and then immediately returning to BL (Friedman ANOVAs: IN, χ^2^_n=5_ = 21.82, p = 0.0013; OUT, χ^2^_n=11_ = 6.667, p = 0.3528; no post hoc comparison reached significance). (**c**) Individual percent changes in E_2_ concentration relative to BL at the end of the three sampling periods for IN and OUT birds (for IN birds the line color refers to the color of areas in panel A; Friedman ANOVAs: IN, χ^2^_n=5_ = 10.00, p = 0.0123; OUT: χ^2^_n=11_ = 2.00, p = 0.7778; post hoc *p < 0.05). The baseline amounts of estrogens per sample in this experiment ranged between 0.041 and 0.395 pg per sample (10 µl; Mean ± S.E.M. = 0.099 ± 0.018, n = 16). Panel A is composed of home made drawings onto which probe positions were reported using Graphic for Mac (https://graphic.com/mac/), other panels were created with Graph Pad Prism 8 (https://www.graphpad.com/scientific-software/prism/). All panels were assembled in Graphic.

We next determined whether variations of locally synthesized estrogens can be detected when testosterone, the aromatase substrate, is provided. Birds (n = 16) were retrodialyzed with aCSF containing testosterone (T; 5 ng/ml) for 60 min. Five of the 16 probes were located in an area expressing aromatase (IN): two in the POM, two in the bed nucleus of the stria terminalis (BST) and one in the ventromedial nucleus of the hypothalamus (VMN) (Fig. 2a). The other 11 probes were located in areas that do not express aromatase (OUT). A marked increase in estrogen concentration (more than 50% increase on average) was observed following T retrodialysis in the IN, but not in the OUT group (Fig. 2b). This rise in estrogens was more progressive than after E_2_ injection and only peaked in the second sample (after 60 min) following retrodialysis onset (See Fig. 2 for detail of statistical analyses).

Noticeably, the increase in estrogen concentration was much larger in 2 out of the 5 positive birds. This larger increase had a marked effect on the mean results (panel B) but also visually decreased the magnitude of changes observed in the 3 other cases (panel C), where an increase to more that 150% of baseline was nevertheless observed. A 50% change is still substantial especially when compared to the complete lack of increase in all subjects where the probe was not in an aromatase positive area (OUT cases). The individual differences between the IN subjects very likely relate to the position of the cannula which is documented in panel A of this figure. Without entering too much into a clinical case by case analysis, the 3 low points concern probes located in or near VMN, where aromatase activity is lower than in POM or BST, and one point that is clearly at the edge of POM.

Together, these experiments thus demonstrate that local changes in extracellular estrogen concentration can be detected in the brain of freely behaving quail upon systemic administration of E_2_ (Exp. 1) and that changes following local administration of the aromatase substrate, testosterone, are only detected when the probe is located within a region containing the enzyme (Exp.2). The next experiments then investigate whether the local concentration of extracellular estrogens also fluctuates in biologically relevant contexts.

### Experiment 3: sexual interactions

To assess whether local concentrations in estrogens changed in response to sexual interactions, males (n = 60 in 4 different cohorts) with a microdialysis probe aimed at a brain region containing aromatase were given physical access to a mature female for 30 min. Males in 3 of these cohorts had been exposed to the view of a female behind a Plexiglas partition as part of another experiment (experiment 5), but only entered in the present experiment after a wash-out period of two hours during which estrogen concentration returned to baseline. Samples were collected every 30 min for 60 min before, during and for 180 min after physical interactions.

**Figure 3:**
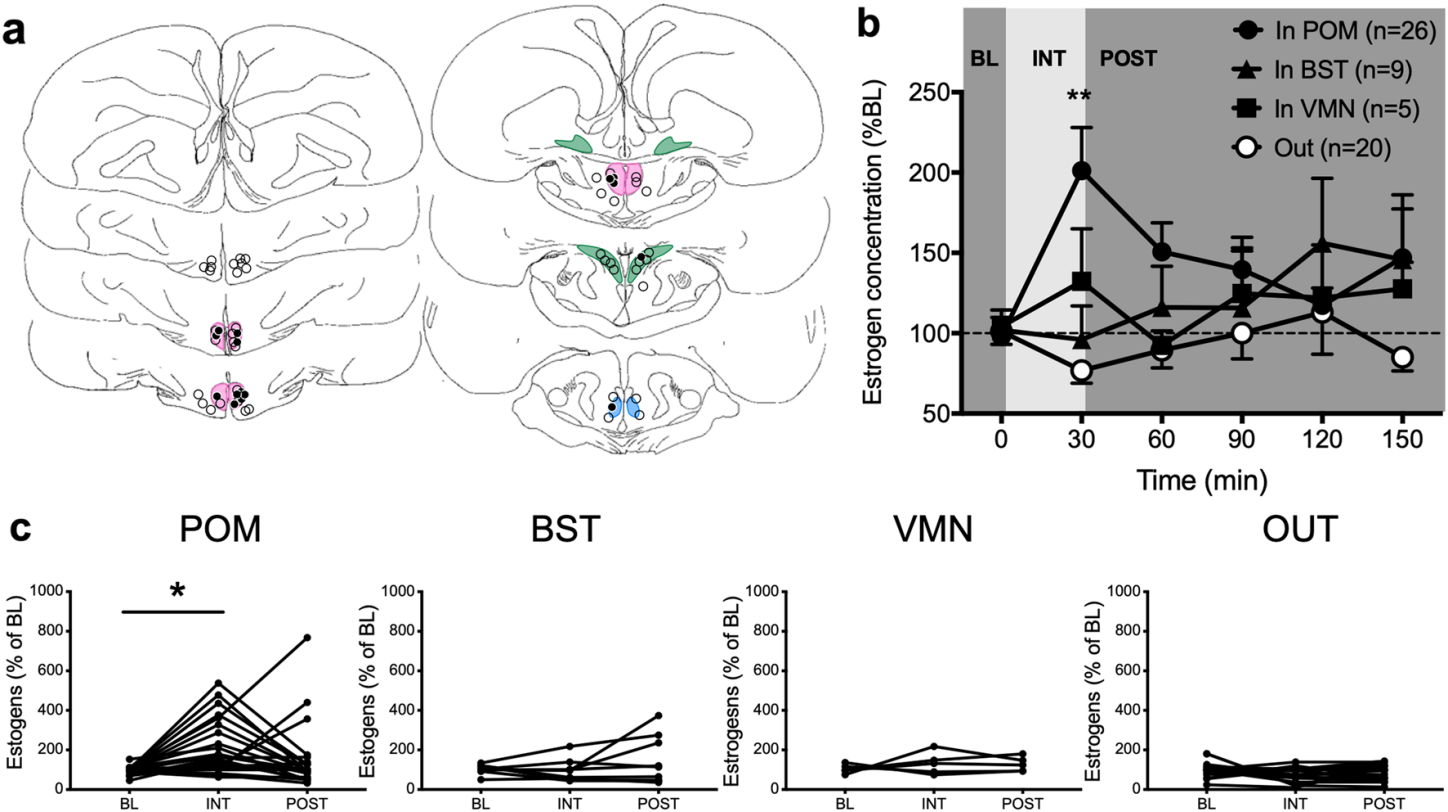
Local estrogen concentrations increase in POM but not in other brain regions during sexual interactions. (**a**) Schematic representation of coronal sections of the quail brain showing the location of the dialysis probes. IN probes are either within or touching the colored zones representing brain regions expressing aromatase. See legend of Fig. 1 for the definition of different brain areas. Black and open circles respectively represent birds in which estrogen concentrations did or did not increase above 150% of the baseline (BL) during the interaction period (last sample of each experimental condition). (**b**) Mean percent changes of estrogen concentration in the dialysate over time following a sexual interaction (based on the mean concentration measured in the last 2 BL samples). OUT: Birds with a cannula outside any aromatase expressing cell populations. INT: physical interaction with the female, POST: post-interaction period. Only males with a cannula in the POM showed a significant rise in estrogens peaking during the interaction period and then gradually decreasing (Friedman ANOVAs: POM, χ^2^_n=26_ = 18.90, p = 0.002; BST, χ^2^_n=9_ = 3.591, p = 0.6097; VMN, χ^2^_n=5_ = 3.2187, p = 0.6664; OUT, χ^2^_n=20_ = 4.4274, p = 0.5100; post hoc **p < 0.01 vs BL). (**c**) Individual percent changes in estrogen concentration relative to BL in the last time point of the three sampling periods for animals whose probe was placed in POM, BST or VMN, or outside a nucleus expressing aromatase (OUT). (Friedman ANOVAs: POM: χ^2^_n=26_ = 10.17, p = 0.0062; BST: χ^2^_n=9_ = 0.7879, p = 0.7065; VMN: χ^2^_n=5_ = 0.4, p = 0.9537; OUT: χ^2^_n=20_ = 5.733, p = 0.0569; post hoc *p < 0.05 vs BL). The baseline amounts of estrogens per sample in this experiment ranged between 0.023 and 1.390 pg per sample (10 µl; Mean ± S.E.M. = 0.201 ± 0.034, n = 60). Panel A is composed of home made drawings onto which probe positions were reported using Graphic for Mac (https://graphic.com/mac/), other panels were created with Graph Pad Prism 8 (https://www.graphpad.com/scientific-software/prism/). All panels were assembled in Graphic.

Probes were again found in different brain areas (Fig. 3a): 26 were in POM, 9 in BST, 5 in VMN, and 20 outside of brain areas expressing aromatase. During the period of sexual interactions, the frequency and latency of the first occurrence of mount attempts (MA) and cloacal contact movements (CCM) were recorded. All birds but 9 copulated with the female. Among the non-copulators, 7 attempted to mount. The probe of the 2 sexually inactive males was within the POM (n = 1) or the BST (n = 1).

Marked increases in estrogens occurred during the period of interaction with the female and were followed by a gradual decrease over the remaining 150 min. Among the 26 birds with a cannula in the POM, 13 showed an increase in estrogen concentration to at least 150% above their baseline during sexual interactions (Fig. 3b,c). This included 3 birds that only performed MA, but no CCM. The only bird of the POM group that did not perform any sexual behavior at all showed no increase in estrogen concentration. In contrast, few birds of the other groups displayed an increase of estrogen concentration (above 150%) during physical interactions with a female (BST: 1/9; VMN: 1/5; OUT: 0/20; Fig. 3c). No final conclusion should however be derived from data concerning BST and even less VMN, since the number of subjects in these groups was too small to provide sufficient statistical power. These data simply illustrate the idea that effects of sexual interactions are very probably neuroanatomically specific to the POM. Surprisingly, the only bird of the BST group that showed an increase in estrogen concentration while the female was present is the only one who did not display any sexual behavior. These potential group differences are supported by statistical analyses that identified significant changes in the POM group only (see detail in Fig. 3).

The change in estrogen concentration in the POM (% of baseline) was not correlated with the frequency or latency of MA or CCM displayed by males during the period of interaction (MA Frequency: r = 0.2223, p = 0.2855; MA Latency: r = − 0.1898, p = 0.3635; CCM Frequency: r = 0.2099, p = 0.3140; CCM Latency: r = − 0.02151, p = 0.9187). Together these observations suggest that simply being with the female and interacting with her may be enough to induce a rise in local estrogen concentration.

### Experiment 4: visual vs. physical interaction

Experiment 3 indicated that sexual interactions with a female result in an increase of estrogen concentrations specifically in the POM, but this increase was also observed in some males who did not copulate (no CCM), although they displayed MA. We wondered whether physical interactions and copulation with a female were required to affect brain estrogen concentrations or if her mere presence would have the same effect. We also wanted to determine whether changes in estrogen concentrations could be mapped with a better time resolution and therefore collected samples every 10 min.

After a baseline period of 60 min, male quail (n = 14) were allowed to see a female behind a Plexiglas partition for 10 min. The partition was then removed allowing the two birds to interact physically. After 20 min of interaction, the female was removed and the male was left alone for 60 min. During the period of physical access to the female, the frequency and latency of first occurrence of MA and CCM were recorded.

**Figure 4:**
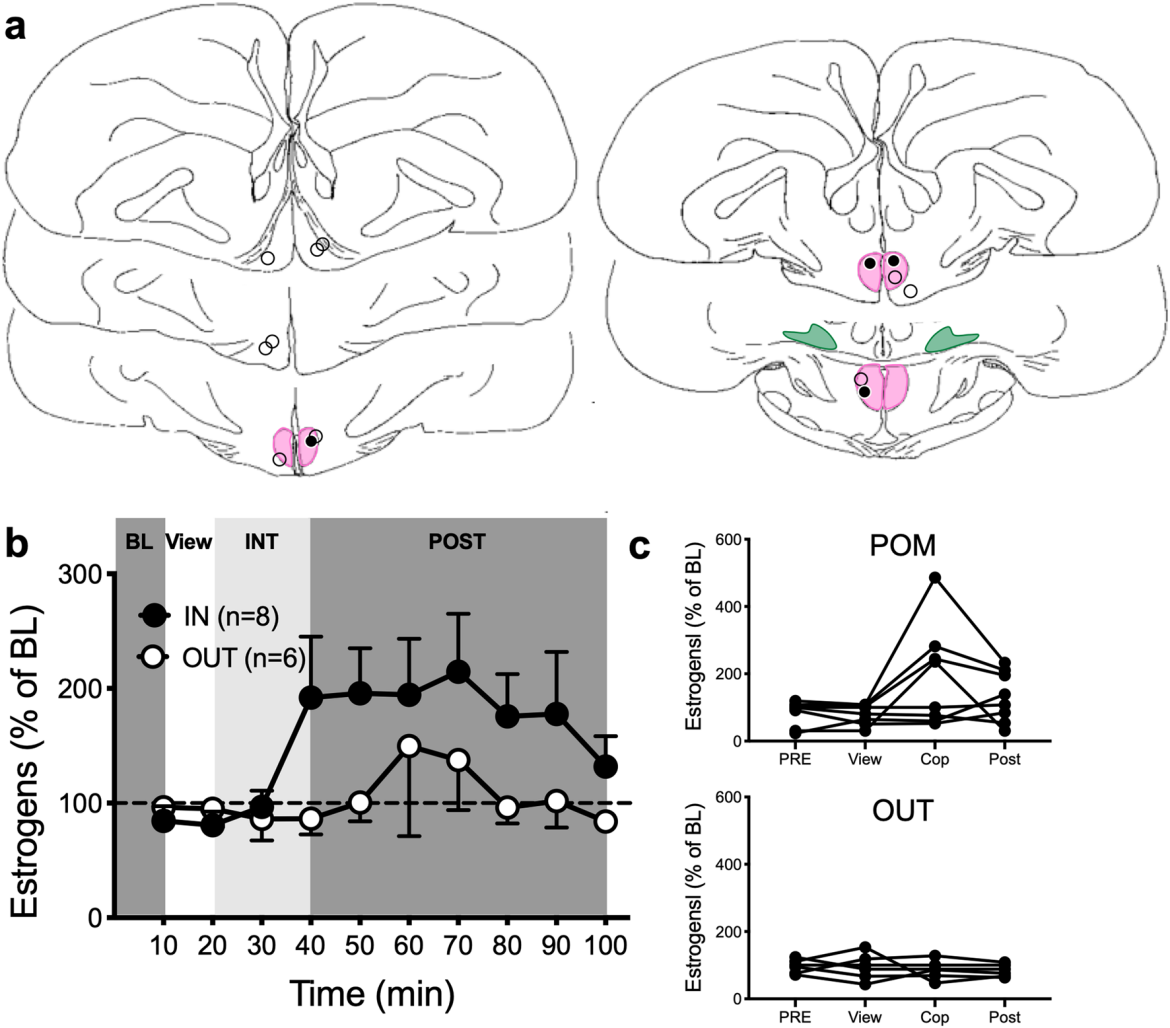
Local estrogen concentrations in the POM increase during sexual interactions but no change is detected in males just after seeing a female for 10 min. (**a**) Schematic representation of coronal sections of the quail brain showing the location of the dialysis probes. IN probes are either within or touching the colored zones representing brain regions expressing aromatase. See legend of Fig. 1 for the definition of different brain areas. Black and open circles respectively represent birds in which estrogen concentration did or did not increase above 150% of the baseline (BL) during the interaction period (last sample of each experimental condition). (**b**) Mean time course of average changes in estrogen concentration in the dialysate as a function of the probe location (based on the mean concentration measured in the last 3 BL samples). Friedman ANOVA: POM, χ^2^_n=8_ = 21.59, p = 0.0103; OUT, χ^2^_n=6_ = 5.418, p = 0.7964. No post hoc comparison reached significance. (**c**) Individual percent changes in estrogen concentration relative to BL in the three sampling periods for animals whose probe was in POM (Friedman ANOVA: χ^2^_n=8_ = 4.629, p = 0.2011, or outside POM (OUT, χ^2^_n=6_ = 1.435, p = 0.7496). View: Period in which the male was allowed to see the female without physical interaction; INT: interaction with a female; POST: Period after the behavioral test. The baseline amounts of estrogens per sample in this experiment ranged between 0.041 and 0.397 pg per sample (10 µl; Mean ± S.E.M. = 0.125 ± 0.019, n = 14). Panel A is composed of home made drawings onto which probe positions were reported using Graphic for Mac (https://graphic.com/mac/), other panels were created with Graph Pad Prism 8 (https://www.graphpad.com/scientific-software/prism/). All panels were assembled in Graphic.

All males copulated when allowed to physically interact with the stimulus female. Their probes were either in the POM (n = 8) or outside any aromatase positive population (OUT; n = 6, Fig. 4a). When the probe was in the POM, no change in estrogen concentration was detected at the end of the 10 min of visual access with the female, but estrogens increased during the physical interaction (more than 150% of baseline in 4 out of 8 males) and remained elevated for the rest of the sampling period. When the probe was outside this nucleus no change of this magnitude in estrogen concentration was detected during the sexual interactions (Fig. 4b,c; See Fig. 4 legend for detail of statistical analyses).

It is important to note that samples were collected here every 10 min, allowing a better time resolution than in the previous experiment. With this shorter periodicity, the increased average concentration of estrogens was fully maintained in POM for 30 min after the removal of the female and had almost returned to baseline 60 min later. This appears to be slightly longer than observed in experiment 3 which was performed with a sampling every 30 min. This sampling frequency however did not permit to know exactly when the peak occurred and what was its true magnitude. The two sets of data are thus very likely consistent.

Together, these results suggest that a rise in local estrogen concentrations can already be detected 20 min after the release of the female. These changes seemed to be specific to the sexual interactions and were not apparently induced by the simple view of a female. However, based on the properties of our probes evaluated in vitro (see Supplementary Information SI2), it is also possible that changes detected here during the period of sexual interaction could have actually been initiated when males were only seeing the female. The next experiment was conducted to tease these possibilities apart.

### Experiment 5: visual access to a female

After a baseline period of 60 min, males (n = 49) were allowed to view a female during 30 min before being left alone for 60 min. Samples were collected every 10 min. The frequency of rhythmic cloacal sphincter movements (RCSM), a measure of sexual motivation in male quail^34^, was recorded during the visual access to the female.

**Figure 5:**
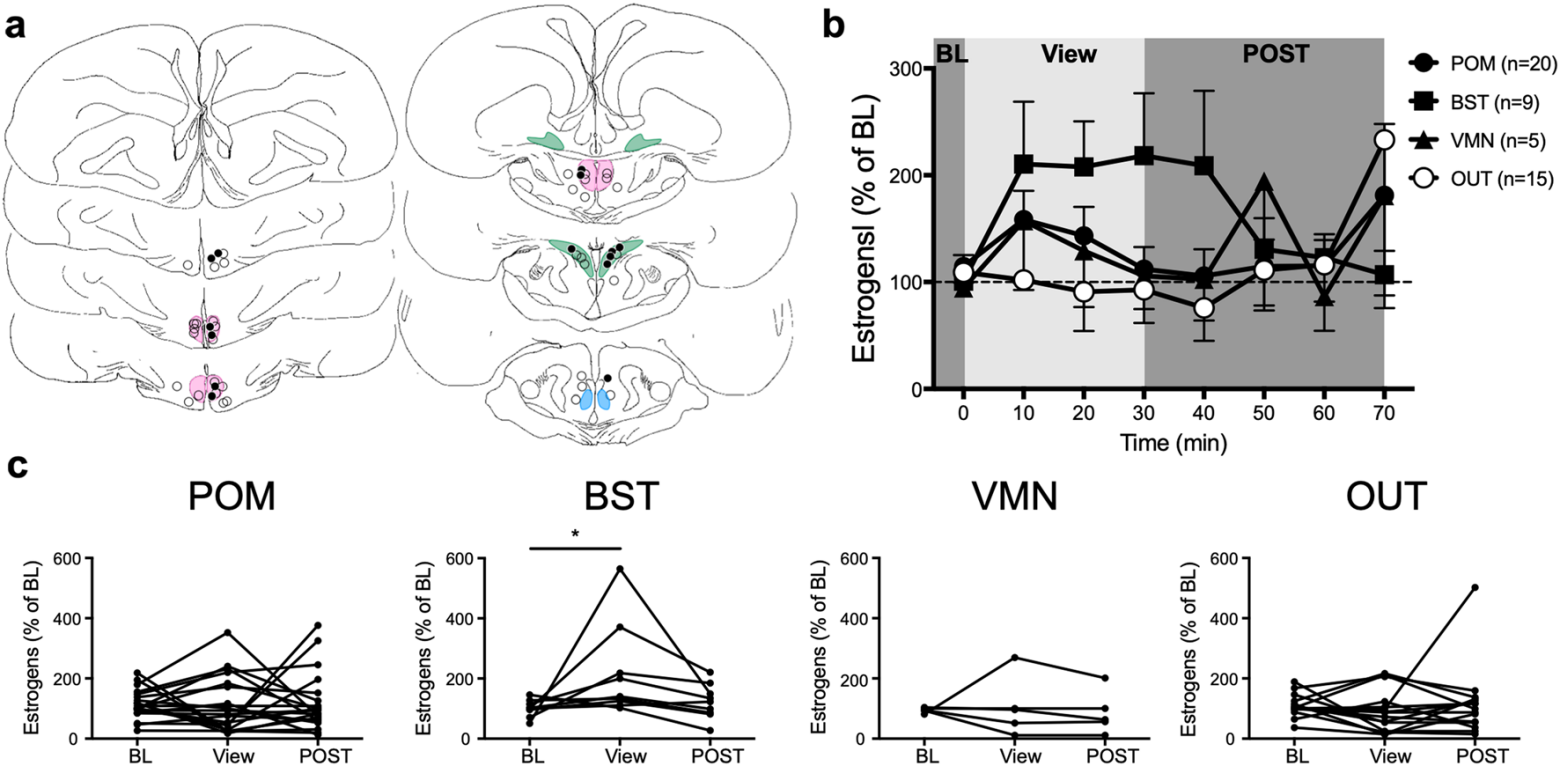
Visual access to a female increases estrogen concentrations in the BST. (**a**) Location of the dialysis probes. IN probes are either within or touching the colored zones representing brain regions expressing aromatase. See legend of Fig. 1 for the definition of different brain areas. Black and open circles respectively represent birds in which estrogen concentrations did or did not increase above 150% of the baseline (BL) during the visual interaction period (last sample). (**b**) Time course of the mean percentage of estrogen concentration in the dialysate (based on the mean concentration measured in the last 3 BL samples). Friedman ANOVA: POM, χ^2^_n=20_ = 9.241, p = 0.2358; BST, χ^2^_n=9_ = 14.69, p = 0.0402; VMN, χ^2^_n=5_ = 13.28, p = 0.0655; OUT, χ^2^_n=15_ = 13.15, p = 0.0686. No post hoc comparison reached statistical significance. (**c**) Individual percent changes in estrogen concentration relative to BL in the three sampling periods for individuals whose probe was in the POM, BST, VMN, or outside any nuclei expressing aromatase (OUT). Friedman ANOVA: POM: χ^**2**^_n=20_ = 1.532, p = 0.4649; BST: χ^**2**^_n=9_ = 7.0, p = 0.0249, No post hoc comparison reached statistical significance; VMN: χ^**2**^_n=5_ = 6.000, p = 0.0694; OUT: χ^**2**^_n=15_ = 0.3415, p = 0.8430. View: Period when the male has visual access to the female, POST: Period after the behavioral test. The baseline amounts of estrogens per sample in this experiment ranged between 0.026 and 1.463 pg per sample (10 µl; Mean ± S.E.M. = 0.159 ± 0.027, n = 49). Panel A is composed of home made drawings onto which probe positions were reported using Graphic for Mac (https://graphic.com/mac/), other panels were created with Graph Pad Prism 8 (https://www.graphpad.com/scientific-software/prism/). All panels were assembled in Graphic.

Probes were located in different populations of cells expressing aromatase (Fig. 5a) including the POM (n = 20), BST (n = 9) and VMN (n = 5) or in brain areas that do not express aromatase (OUT, n = 15; Fig. 5a). As previously described^35,36^, visual access to the female led to a drastic increase in the frequency of RCSM independently of the probe location (Baseline 3.35 ± 0.78, view 256.70 ± 21.87; n = 40; t_78_ = 11.57, p < 0.0001).

Visual access to a sexually mature female led to an increase in estrogen concentration in the BST. This rise was detected within 10 min of seeing the female, lasted for the whole period of visual access and then rapidly returned to baseline values once the female was removed (see Fig. 5b for details and post hoc comparisons). No correlation was observed between the RCSM frequency during visual access to the female and the increase in estrogens observed in the BST (r = − 0.008, p = 0.9593).

It is important to note again that there were relatively large individual differences in this experiment, but one main point could be established: visual access to a female behind a glass partition triggers an increase in estrogen concentrations in the BST. As also observed in experiment 2, in one male with a probe in BST estrogen concentration increased to 600%, which markedly increased the overall mean, but also flattened the visual representation of all other birds in the group, although these nevertheless experienced substantial increases. Overall these changes resulted in a statistically significant difference, which would not be the case if most birds were not showing a change in the same direction. All variations in concentration in other nuclei were not systematic nor were they statistically significant (Fig. 5c; see legend of Fig. 5 for detail of statistical analysis). They are presented here only for illustrative purposes.

Together, these results confirm that changes in the local concentration of brain estrogens can be detected as fast as within 10 min and more importantly reveal that seeing a female behind a glass partition results in a different pattern of local endocrine changes compared to the pattern observed when the male is allowed to physically interact with her.

### Experiment 6: first minutes of sexual interaction

In sexually experienced Japanese quail, copulation occurs very quickly (10 to 20 s) after a female becomes available^37^. We wondered whether changes in estrogen concentrations induced by sexual interactions potentially take place after such short latencies (faster than 10 min observed in previous experiments). To investigate this question, a subset of birds from experiment 5 (n = 13) remained in the microdialysis chamber overnight with continuous aCSF perfusion. On the next morning, after 60 min of baseline sampling, they were given physical access to a female for 24 min and then left alone for 120 min. Samples were collected after 2 and 4 min and then every 10 min until the end. Seven of these birds were bearing a probe in the POM (POM), whereas 6 birds had a cannula outside of any aromatase population (OUT).

**Figure 6:**
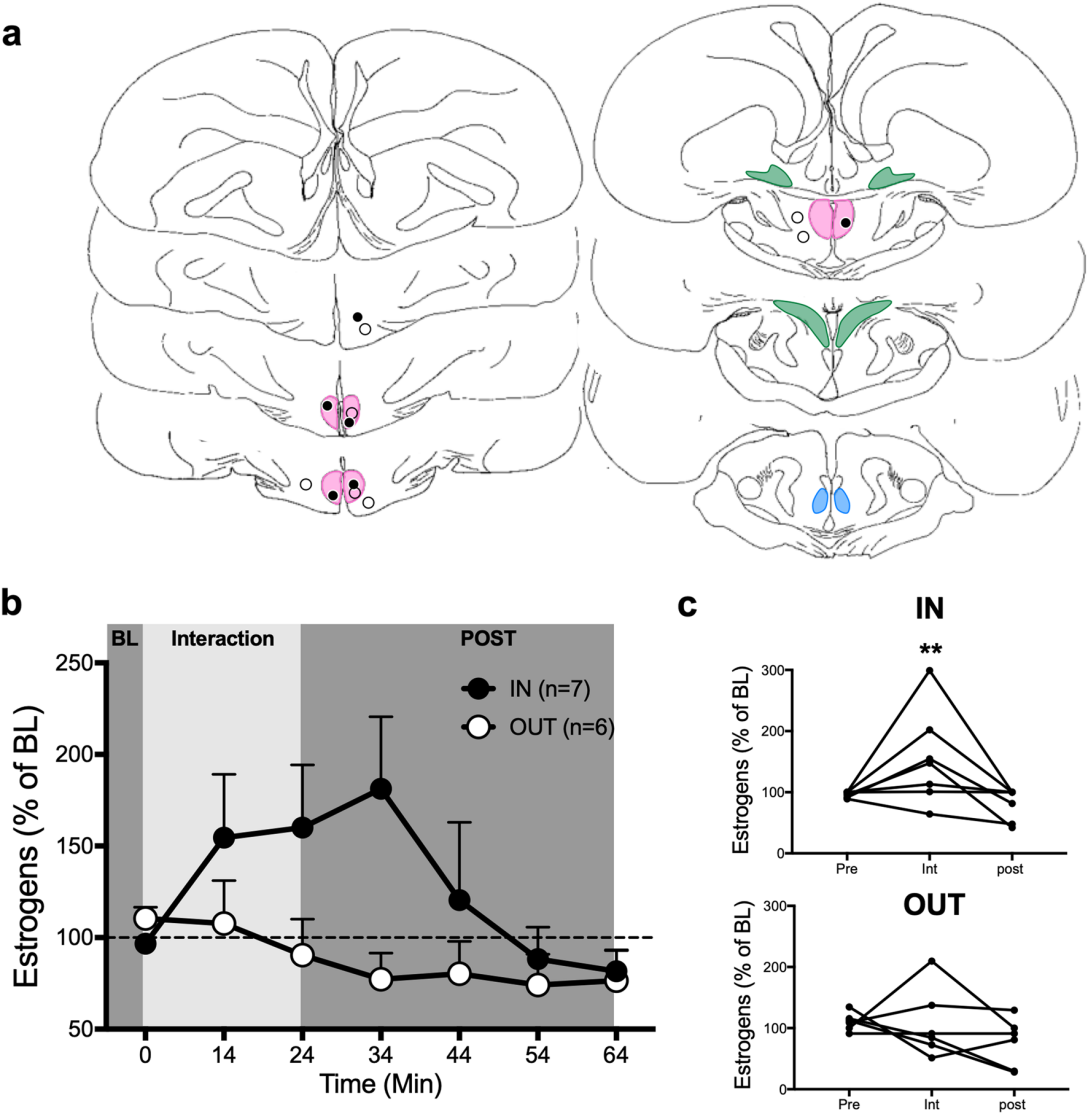
Changes in local estrogen concentration are detected within 10 min of sexual interaction in medial preoptic nucleus (POM) but not outside. (**a**) Schematic representation of coronal sections of the quail brain showing the location of the dialysis probes. IN probes are either within or touching the colored zones representing brain regions expressing aromatase. See legend of Fig. 1 for the definition of different brain areas. Black and open circles respectively represent birds in which estrogen concentrations did or did not increase above 150% of the baseline (BL) during the interaction period. (**b**) Mean time course of changes of estrogen concentration in the dialysate during the course of the experiment expressed as the percentage of estrogen change based on the mean concentration measured in the last 3 BL samples. BL represents the last point of the 60 min baseline period during which samples were collected every 10 min. IN: Birds with the probe in POM, OUT: Birds with the probe outside any aromatase expressing cell populations. POST: post-interaction period. Friedman ANOVA: IN, χ^2^_n=7_ = 19.58, p = 0.0033; OUT: χ^2^_n=7_ = 5.837, p = 0.4417. No post hoc comparison reached significance. (**c**) Individual percent changes in estrogen concentration relative to BL in the three sampling periods for animals whose probe was within or outside POM. Friedman ANOVA (POM: χ^2^_n=7_ = 6.348, p = 0.0501; OUT: χ^2^_n=6_ = 0.9774, p = 0.6559; post hoc : **p < 0.01 vs POST). The baseline amounts of estrogens per sample in this experiment ranged between 0.053 and 0.315 pg per sample (10 µl; Mean ± S.E.M. = 0.147 ± 0.024, n = 13). Panel (**a**) is composed of home made drawings onto which probe positions were reported using Graphic for Mac (https://graphic.com/mac/), other panels were created with Graph Pad Prism 8 (https://www.graphpad.com/scientific-software/prism/). All panels were assembled in Graphic.

All but 2 samples (out of 26) collected after 2 or 4 min of sexual interactions were below the detection limit of the assay. However, the mean estrogen concentration in samples collected 10 and 20 min later was higher than during baseline in the POM group (see Legend of Fig. 6 for detail of statistics).

Friedman analyses on the last point of baseline, interaction and post condition revealed a significant effect and a strong trend of change in estrogen concentration across experimental conditions only in the POM group. Post hoc tests showed that estrogen concentration was higher in the POM when the female was present than during the period that followed (Fig. 6). The return to baseline took 20 to 30 min depending on whether we take the end of the interaction or the point 10 min later as starting point. This might be slightly faster than the 30 to 60 min observed in experiments 3 and 4, although comparison with experiment 3 remains difficult since it was based on a less frequent sampling. The source of this potential difference is difficult to identify, but since these experiments were run over a period of 7–8 years the slight differences in the conditions in which the experiments were run may contribute to this variability. Overall data remains however quite comparable.

### UHPLC-mass spectrometry assays

Ultra-high performance liquid chromatography coupled with electrospray ionization tandem mass spectrometry (UHPLC-ESI–MS/MS) was also used to confirm the nature of the steroid whose concentration increased locally in the brain during sexual interactions. These assays demonstrated that concentrations of E_2_ measured by RIA correlate with concentrations measured by UHPLC, but these concentrations were systematically lower in the latter technique. UHPLC also showed that a large number of estrogens and their metabolites are present in the dialysates and that E_2_ only represents 19.6% of the total. The differences observed between the two techniques could therefore relate either to a systematic technical imprecision or to the fact that the RIA actually detects other estrogens besides E_2_ (See Supplementary information SI3).

## Discussion

We demonstrate here that rapid changes in brain estrogen concentration can be monitored by in vivo microdialysis coupled to an ultra-sensitive RIA with a time resolution of 10 min. Extracellular estrogen concentration increases in the POM, but apparently not in the BST, VMN or adjacent populations of cells expressing aromatase within 20 min during sexual interactions with a female. This rise lasts for 30 to 60 min after the female has been removed. Visual access to a female also leads to an increase in estrogens in the BST within 10 min, but not in the other regions investigated. This represents the fastest changes in local estrogen concentration ever reported in vivo.

One could speculate as to why birds that were allowed to freely interact with a female did not exhibit in Experiment 3 an increase of estrogen concentration in the BST, as demonstrated in Experiment 5, since they also obviously saw the female. There are several possible explanations for this apparent discrepancy. First, the timing of sample collections was different between these two experiments (every 30 vs. 10 min). The increase of estrogens in BST after viewing the female lasted about 40 min in Experiment 5 and could therefore have concerned one single data point only (the 30 min time point) in Experiment 3. The effect could thus have been missed easily if the timing of the response in this different context (see next 2 points) was slightly different. Second, and more importantly, it must be considered that seeing a female that is inaccessible behind a glass partition is a very different situation from accessing her immediately and engaging in copulatory behavior. The enhanced motivation and associated frustration of seeing this female that cannot be reached could clearly be part of the neural activity induced by the situation in BST. Third, the behavior of the female in these two contexts is also probably very different and therefore potentially induces different neuronal reactions. And forth, one should consider that engaging in copulatory behavior, which clearly activated the release of estrogens in the POM, might also inhibit this release in BST through the bidirectional connections that are known to link these two nuclei. We obviously cannot discriminate among these different interpretations, but the two sets of results are clearly not incompatible.

Somewhat surprisingly the increases in estrogens did not correlate with the frequency of sexual behaviors, nor with the frequency of RCSM, and one bird who exhibited no sexual behavior still displayed an increase in estrogens during experiment 3, suggesting that these increases do not reflect the motor component of the behavioral interaction but rather relate to its sensory or motivational aspects.

### Technical challenges

These studies were only possible thanks to the highly sensitive RIA allowing the detection of estrogen concentrations well below 1 pg/10 µl of sample. Concentrations measured here fluctuated between 0.026 and 1.461 pg/sample and would not be detected by standard assays. Note also that brain concentrations must clearly be higher since steroids are liposoluble; thus they poorly dissolve in an aqueous aCSF solution and exchange rates between the extracellular milieu and the dialysate are not sufficient to reach equilibrium at least with the flow rate of 1 µl/min used here. In vitro validations revealed that after 30 min, E_2_ concentration inside the probe is only 10–15% of the concentration outside the probe (See Supplementary Information SI2). The fastest significant increases in estrogens were detected after 10 min of interaction with a female, but samples collected after 2 min failed to detect a significant change. Given these dynamics of exchange between the external media and the dialysate, it is likely that changes in the brain, in particular within neurons expressing aromatase, occur much faster. Locally produced estrogens need to diffuse outside the neurons into the extracellular milieu and then into the dialysate in sufficient concentrations to be detected.

The RIA and dialysis procedures used in the present experiments were submitted to extensive validations. This assay was demonstrated to track both in vitro (Supplementary Information SI2) and in vivo (Experiment 1) E_2_ changes in the external compartment, and it only detected a change in estrogens following testosterone retrodialysis, if the probe was inside an aromatase-positive brain region (Experiment 2). The RIA itself is based on an antibody that exhibits high specificity for E_2_ according to the manufacturer’s instructions. Intriguingly, substantial differences were detected between assays performed by RIA and UHPLC-MS (See Supplementary Information SI3). This discrepancy might potentially reflect detection by the RIA of additional compounds besides E_2_, but we remain confident that the results presented here truly concern changes in E_2_, or at least of an estrogen or an estrogen metabolite derived from local aromatization. It is notable that UHPLC-MS detected high concentrations of 2OH-E_1_ in the dialysates which is consistent with a previous experiment in quail demonstrating the presence of a very active estrogen 2-hydroxylase in the quail POM^38^.

One must also acknowledge that the average estrogen changes identified here were associated to large individual differences. A large part of these differences presumably relates to differences in probe location, but may also reflect genuine individual differences in the endocrine physiology of the subjects or in their responses to the test environment/conditions or the cues from the females (see Supplementary Information SI1).

### Relationships with brain aromatase activity

As AA is broadly considered as a proxy for fluctuations in estrogen concentration, we wondered how these rapid increases in estrogens are compatible with the rapid decreases in brain AA previously identified after sexual interactions with a female. Indeed, we previously demonstrated that brain AA measured ex vivo significantly decreases 2 to 5 min after a male has interacted sexually with a female^26,27^, while increases in AA are observed at similar latencies after exposure to an acute stress^39,40^.

This discrepancy is probably explained by the fact that estrogens and AA have been assayed at different time points relative to behavioral expression (after or during behavior) and that AA is measured ex vivo in assay conditions (optimized concentration of co-factor and pH, homogenized tissue putting in contact cells compartments that are normally separated, …) that might not reflect the enzymatic activity in vivo^28^. Based on all available evidence, the most likely interpretation of these data is that AA rapidly increases at the beginning of the interaction with the female, which almost immediately results in an increased estrogen concentration in the brain. In a second step, AA would then decrease below its baseline level due to a feedback mechanism that remains unidentified. Two main options are currently most plausible. First, AA is rapidly reduced by the release of glutamate induced by the interaction with the female. Glutamate was indeed shown to be released in the POM within 3 min after copulation with a female^41^ and it also inhibits brain AA both in vitro^23^ and in vivo^41^. Alternatively, the release of neuroestrogens as a consequence of an increased AA might involve, as it is the case for neurotransmitters, the apposition and opening of presynaptic vesicles containing aromatase^42^ to the synaptic membrane. This would putatively expose the enzyme to the extracellular milieu and this new micro-environment would inhibit its activity^22,24^. AA is indeed known to be drastically inhibited in vitro by increased concentrations of Ca^2+^, a feature of the extracellular medium^43^. Although the lipophilic steroids are supposed to diffuse freely through cells and membranes, there is evidence in drosophila that the release of the steroid hormone ecdysone is mediated through a calcium-regulated vesicular trafficking mechanism^44^. Such a scenario is therefore not impossible.

### Behavioral significance

What could then be the functional significance of such rapid changes in estrogens given that a sexually experienced male quail can achieve full copulation within a few seconds of its introduction to a female^37^? Could they modify ongoing behavior, reinforce sexual motivation or affect learning to facilitate future behavior?

We previously showed that increasing or decreasing the estrogen concentration in the quail brain via a single i.c.v. injection of E_2_ or of an aromatase inhibitor respectively increases or decreases within 15 to 30 min sexual motivation as assessed by the frequency of RCSM^16,45^. This latency presumably overestimates the time needed for E_2_ to act due to technical limitations related to the time needed for diffusion to the active sites. Current technical limitations thus still prevent us from assessing both how quickly estrogens change in the brain and how quickly these changes potentially affect behavior.

In the auditory cortex of zebra finches, it was shown by in vivo microdialysis that estrogen concentrations vary with a latency of 30 min after exposure to social stimuli^32^, but importantly electrophysiological studies indicated that changes in E_2_ local concentration have almost immediate effects on the processing of auditory information, in particular songs of conspecifics^46–48^. It is therefore quite conceivable that estrogens increase within seconds in the relevant neurons of the quail brain when a male is exposed to a female and that this increase enhances just as rapidly the processing of sexual stimuli (visual, auditory, acoustic?) originating from this female. The increased salience of sexual stimuli would focus the attention of the male to the female and increase the probability of the male engaging in sexual behavior.

At the phenomenological level, this is essentially the equivalent of an increase in motivation. In both rats and quail, dopamine is released in the preoptic area in response to a female and this response predicts whether the male will or will not go on to copulate^49–51^. Estrogens acutely potentiate dopamine release in the mesolimbic system^52^. It is thus possible that the increase in local estrogen concentration observed in the POM during sexual encounters potentiates the DA response to the female thus facilitating copulation. Regarding the changes in estrogens observed in the BST following visual exposure to a female, recent work in mice revealed that aromatase neurons of the principal component of the BST are active during sexual interactions and might play a role in sex recognition^53^. However, whether this role relies on estrogen synthesis is not known. The present results are congruent with the idea that the activity of aromatase neurons is enhanced in the BST during the early events of sexual interactions resulting in increased estrogen synthesis. Whether the produced estrogens play a role in sex partner preference remains, however, to be tested.

Alternatively, in the quail POM estrogens could also rapidly modulate premotor outputs. Indeed, it has been shown that in plain midshipman fish (*Porichtys notatus*) E_2_ increases within 5 min the activity of a hindbrain-spinal pacemaker circuit that directly establishes the fundamental frequency and duration of vocalizations^54^.

Finally, these local increases in estrogens could have no immediate function but modulate the acquisition and storage of information related to the female and to sexual interaction that would facilitate subsequent copulatory behavior. Although male sexual behaviors is considered to be an innate behavior, copulatory efficiency increases with experience^55,56^. There is extensive evidence demonstrating that (neuro)estrogens modulate memory processes and some of these effects take place quite rapidly after exposure to estrogens^57,58^. Estrogens are also implicated in the control of reward processes in females^59,60^ and similar actions in males could enhance the rewarding value of sexual stimuli in males. Interestingly, the BST, one nucleus where estrogens increase following visual exposure to a female, is well positioned to play such a role because it is at the interface of the Social Behavior Network and of the mesolimbic reward system^61^. The increase in estrogen concentration observed in the BST could thus be important to integrate sexual stimuli, evaluate their salience, and correctly respond to them.

In conclusion, this study demonstrates that it is possible to measure local changes in steroid concentration within 10 min in freely behaving quail and reveals that local estrogen concentrations are tightly regulated over time in behaviorally relevant contexts. The temporal-, spatial- and context-specificity of these changes is compatible with a neuromodulatory nature of neuroestrogens^62,63^. Future work should now evaluate how broadly these rapid changes in brain estrogens occur, in terms of species diversity and multiple brain sites, and what are their specific behavioral consequences.

## Material and methods

### Subjects

The study complied with the ARRIVE guidelines. This study was performed on male Japanese quail (*Coturnix japonica*), a well-known animal model in behavioral neuroendocrinology^34^. A total of 96 males were obtained from a local breeder or from our breeding colony at the University of Liège (Animalerie Centrale de l’ULiège, GIGA Neurosciences, agreement number: LA1610002). Some of these males were used in two successive experiments after a wash-out period of at least two hours during which E_2_ concentrations returned to baseline. A total of 64 females were also used as stimuli. Birds were raised in mixed-sex groups until the age of approximately 6 weeks, when they were moved to individual cages. Throughout their life in the lab, they were maintained under a photoperiod simulating long summer days (16L: 8D) and provided with food and water ad libitum. All experimental procedures were in agreement with the Belgian laws on the “Protection and Welfare of Animals” and on the “Protection of Experimental Animals”. The protocol was approved by the Ethical Committee for the Use of Animals of the University of Liège (protocol #1442). Surgeries were carried out under gas anesthesia (isoflurane [Isovet, Verdifarm] in oxygen; see Supplementary Information SI1). Animals were euthanized by rapid decapitation under 5% isoflurane or perfusion under irreversible anesthesia (Euthasol®, 120 mg/kg; see Supplementary Information SI1).

### Experimental design

The in vivo microdialysis procedure was adapted based on a previous in vivo microdialysis study on quail^41^. For details about the stereotaxic surgery and microdialysis procedure, see Supplementary Information SI1.

For all experiments except experiments 1 and 2, males were sexually experienced (see Supplementary Information SI1 for details). For all experiments except experiments 4 and 5, the arena was a glass aquarium of 50 cm (length) × 30 cm (width) × 40 cm (height). For experiments 4 and 5, this aquarium was divided by a removable glass partition in two compartments (one of 35 cm in length containing the experimental male, the other of 15 cm in length containing a stimulus female) such that the male had visual, but not physical, access to the female.

Each experiment was divided into three sampling periods during which samples were collected every 30 (Exp 1–3), 10 min (Exp 4–5) and 2 or 10 min (Exp 6). During the baseline period the male remained alone in the arena. During the experimental period the male was exposed to either a pharmacological treatment or a behavioral stimulus. The post-experimental period started when the stimulus was removed and the male remained alone and undisturbed in the arena. Depending on the experiment, the baseline period lasted 60 to 90 min (with the exception of Fig. 1) of which the last 2 or 3 samples were used to calculate an average baseline value for each individual that was then used to calculate the percent changes over time for each animal. At the end of the experiment, brains were collected to check the anatomical position of the cannula. Six independent experiments were carried out with this procedure.

### Estrogen measurements

Microdialysates were assayed for estradiol by commercially available ^125^Iodine-E2 radioimmunoassay kits (DSL-4800, Ultra-sensitive Estradiol RIA, Beckman Coulter; see Supplementary Information SI1). A fraction of the samples collected during Experiment 3 was also assayed by ultra-high performance liquid chromatography coupled with electrospray ionization tandem mass spectrometry (UHPLC-ESI–MS/MS) in an attempt to further confirm the specificity of the E_2_ RIA (Supplementary information SI3). Although the manufacturer reports minimal cross reactions of the antibody with estrogens other than estradiol, RIA does not allow for an unambiguous identification of the estrogen measured but provides a global measure of estrogens^64^. All measures obtained by RIA in this study are thus reported as “estrogens”.

### Statistical analyses

All statistical analyses were performed by non-parametric methods using GraphPad Prism 8 due to non-normal distributions and/or significant differences in variance between groups. Except for experiment 1 where real concentrations were used, all data were expressed as the percentage of the last 2 or 3 points of the baseline for each subject to accommodate individual differences in absolute concentration.

## Supplementary Information

Supplementary Information.

## Data Availability

The file containing the raw data is uploaded and available here: 10.6084/m9.figshare.14207945.
