# Supplementary material for: Rapid changes in brain estrogen concentration during male sexual behavior are site and stimulus specific
Source: Sci Rep. 2021 Oct 11;11:20130. doi: 10.1038/s41598-021-99497-1 (PMC8505645; doi:10.1038/s41598-021-99497-1)
Supplement: Supplementary file 1 — Supplementary Information. [file 41598_2021_99497_MOESM1_ESM.pdf]

Supplementary material

Rapid changes in brain estrogen concentration during male sexual behavior are site and stimulus specific

Marie-Pierre de Bournonville[1]†, Catherine de Bournonville[1]†, Laura M. Vandries[1], Gwenaël Nys[2], Marianne Fillet[2], Gregory F. Ball[3], Jacques Balthazart[1], Charlotte A. Cornil[1]

Supplementary information SI1 : Additional details on methods

Surgical procedures

At the age of approximately 8 weeks, birds were stereotaxically implanted under gas anesthesia (isoflurane [Isovet, Verdifarm] 5% and 2% in oxygen for induction and maintenance respectively) with a microdialysis guide cannula (Microbiotech/se, Stockholm Sweden, MAB4.15.IC, 22 gauges) targeting the medial preoptic nucleus (POM). Birds were placed in a stereotaxic apparatus with the beak holder aligning the horizontal axis of the brain with the horizontal axis of the stereotaxic apparatus. After the skull was opened, the guide cannula was lowered to the targeted location and then secured to the skull using dental cement. Coordinates of the tip of the cannula were 1.6 mm anterior (X), 2.5 mm dorsal (Y) and 0.5 mm lateral (Z) to the zero point (center of inter-aural axis). A dummy 300 µm longer than the cannula was then inserted in the guide cannula to seal it until the day of the experiment.

Sexual experience

After one week of recovery, males were fitted with a harness that they kept until the end of the experiment. After one week of habituation to the harness, males were given the opportunity to gain sexual experience (pre-test) for a subset of experiments (experiments 4-6). They were placed in a testing arena (60 cm [length] x 40 cm [width] x 50 cm [height]) in the presence of a sexually mature female for 5 min, every day until they showed the copulatory sequence within 10 sec of test onset. This was achieved after a minimum of 5 pre-tests. During the last pre-test, males were tethered to the balanced arm and connected to the collection tubing (see next section) to habituate them to the experimental device and they were again allowed to freely interact with the female in the pre-test arena. The experimental device did not affect the copulatory ability of the males. All birds were also habituated to the empty microdialysis arena for a minimum of one hour on a day when they were not in contact with a female. These habituation procedures avoided establishing any association between the dialysis arena and sexually relevant stimuli.

***In vivo* microdialysis procedure**

The procedure was adapted based on a previous *in vivo* microdialysis study on quail (de Bournonville *et al.*, 2017). Prior to insertion into the guide cannula, microdialysis probes (MAB4.15.2.PES, Microbiotech/se; outer diameter 0.2 mm, length 1.8 mm, 6 kDa cutoff) were perfused for approximately 5 min with artificial cerebro-spinal fluid (aCSF; 199 mM NaCl, 2.5 mM KCl, 1 mM NaH_2PO_4 [monohydrate], 1.3 mM $MgSO_4$ [heptahydrate], 2.5 mM $CaCl_2$ [dihydrate], 11 mM glucose, 26.2 mM $NaHCO_3$, 1% bovine serum albumin, pH=7.2-7.4) at a speed of 2 µL/min, while immersed in aCSF. The probe was then inserted into the guide cannula, the flow rate was decreased to 1 µL/min, the bird was placed in the dialysis arena and left alone overnight (or for 4 h in experiment 4) in order to habituate to the perfusion prior to collection of the first sample.

Probes were connected to a Hamilton syringe (Gastight, 2500 µL, Sigma-Aldrich) placed in an infusion pump (KDS 220 Multi-Syringe Infusion Pump) using FEP tubing (Microbiotech/se, 4001005; ID: 0.15 ± 0.5 mm, OD: 0.4 mm). To avoid entangling, the tubing was attached to a swivel mounted on a balanced arm fixed above the arena. To remove any tension on the bird's head and prevent tension in the tubing due to the animal's movements, males were tethered to the balanced arm by their harness and the tubing was attached to it.

Brain collection
Depending on the experiment, brains were either fresh frozen, fixed in acrolein (5%, in Phosphate Buffered Saline [PBS], Sigma-Aldrich 110221), or fixed by perfusion with paraformaldehyde (PFA, Sigma-Aldrich, 4% in PBS). For fresh frozen brains, India ink was inserted in the guide cannula using the dummy cannula whose tip had been dipped in ink and birds were euthanized immediately after by rapid decapitation under isoflurane 5%. Brains were then collected and directly frozen on dry ice. For fixed brains, birds were either perfused through the heart under irreversible anesthesia (Euthasol®, 120 mg/kg) with saline (NaCl 0.9%, 400 mL), followed by PFA (500 mL), or euthanized by rapid decapitation under isoflurane 5%, their brain was collected, immersed in acrolein for 2.5 hours, and washed twice in PBS for 30 min. Fixed brains were then cryo-protected in sucrose (30%, in PBS) for 2 days, frozen on dry ice and stored at -80°C until they were sectioned on a cryostat. Alternate sets of sections were then Nissl-stained or stained by immunohistochemistry for aromatase.

Histology and probe location
Brains were sliced into 30 µm-thick coronal sections (from the level of the tractus septopallio-mesencephalicus [TSM] to the caudal end of the hypothalamus) with a cryostat at -20°C. Fresh frozen brain sections were collected on superfrost® slides and Nissl-stained. Briefly, sections were first rehydrated in successive baths of decreasing alcohol concentration (Isopropanol 90%, 70%, 20%, ddH2O) for 1 min each. They were then immersed in toluidine blue (200 mg/100 ml of Walpole buffer) for 1 min, followed by 2 rinses in Walpole buffer of 15 min each. Staining was fixed in a solution of molybdate (5g/100ml, in H2O) for 2.5 min. Sections were then dehydrated using 6 successive baths of increasing alcohol concentration (ddH2O, Isopropanol 20%, 70%, 90%, 100%, 100%) for 1 min each and finally cover-slipped using Eukitt (Sigma-Aldrich, 03989). The probe location was determined using both location of ink during brain slicing at the cryostat and the location of the tip of the cannula as identified under a microscope.

Fixed brains were cut and collected in 4 series in PBS. One of them was Nissl-stained as described before and another one was immunostained for the enzyme aromatase using a rabbit primary polyclonal antibody raised against recombinant quail aromatase (QR2/05) that was previously validated for use in quail brain (Foidart et al., 1995; Carere et al., 2007). Immunohistochemical labeling was carried out by the avidin-biotin technique on free-floating sections, as previously described after acrolein fixation (Bardet et al., 2010). Briefly, sections were incubated successively in sodium borohydride (0.1% in Tris Buffered Saline [TBS]) for 15 min, in hydrogen peroxide (0.6%, in TBS) for 30 min, and saturated in Normal Goat Serum (NGS; 5%, in TBS with 0.1% of Triton X-100, Sigma-Aldrich [TBST]) for 1h. Each step, but the last, was followed by three 5 min washes in TBS. Sections were then incubated twice overnight in the primary antibody (QR2/05, 1:3000, in TBST) along with NGS 5% at 4°C. After 3 washes, they were incubated with a biotinylated goat anti-rabbit antibody (Jackson 111-065-003, 1:400, in TBST) for 2h. After 3 washes, the antibody-antigen complex was visualized by the avidin-biotin complex method performed with the Vector Elite Kit (Kit ABC Vectastain Elite PK-6100, Vector Laboratories PLC). After three final washes, the peroxidase was visualized with diaminobenzidine (3,3-diaminobenzidine tetrahydrochloride, Sigma-Aldrich, 0.04% along with H_2O_2 0.012%, in TBS) as chromogen.

The probe location was determined by the analysis of both sections stained by Nissl and for aromatase. As anticipated, random errors in cannula placement ended up producing subjects with cannula placed in the 3 main nuclei expressing aromatase of the Social Behavior Network (SBN; (Newman, 1999)): the medial preoptic nucleus (POM), the Bed Nucleus of the Stria Terminalis (BST) and the Ventromedial nucleus of the Hypothalamus (VMN), as well as outside these nuclei (OUT; see results of specific experiments). In all cases, it was found that sufficient numbers of males had a probe correctly located in the POM (or BST) in order to draw statistically supported conclusions. Birds with a probe in other locations were in variable numbers depending on the experiment. The data corresponding to these other probe locations are described in the result section, but they were sometimes not numerous enough to permit drawing firm conclusions. They should thus be considered only as either additional information suggesting the neuroanatomical specificity of the effects observed when probes are in POM or suggestions of effects that might be worth investigating in future work.

Radioimmunoassay of estrogens

Estrogens in dialysates were measured using a commercially available ^{125}Iodin-E_2 radioimmunoassay kit (DSL-4800, Ultra-sensitive Estradiol RIA, Beckman Coulter) essentially following the manufacturer recommendation except that more diluted curve points were added to be able to quantify lower concentrations. The adapted curve ranged between 0.078 and 11.25 pg/tube and the average sensitivity across experiments was 0.059 pg/tube, as determined by values with a B/B0 of 97%.

All samples were run as singletons (except for *in vitro* validations, in which samples were run in duplicates) due to the low concentration of estrogens in the samples and their limited volume (10 to 30 µl/sample). This radioimmunoassay was previously described and validated for quail serum and brain samples (Dickens *et al.*, 2014)

Briefly, diluted samples (300 µl) were incubated at room temperature for 4 h with 100 µl of anti-E2 antiserum (diluted 1:3.5 in Phosphate buffer saline with gelatin [PBSg]). Then 100 µl of ^{125}I-E_2 (diluted 1:3, in PBSg) was added and tubes were incubated at 4°C for 24 h. The precipitating reagent (500 µl) was added and tubes were incubated for 20 min at room temperature, prior to a centrifugation step (1500 g for 20 min, at room temperature). Finally, supernatants were decanted and pellets were counted with a gamma counter. Undetectable values ($B/B_0 \geq 0.97$ in the assay) were fixed at the value obtained for a B/B_0 of 0.97 in the given assay). These very low values were however essentially observed in the baseline samples. When males were exposed to sexual stimuli, estrogen concentration in the dialysates increased and the corresponding B/B_0 in the RIA dropped to values usually ranging between 0.90 and 0.60. The peaks of E_2 that are reported in the present study were thus measured in the optimal linear and most sensitive part of the curve. The small uncertainty related to values that were read near or above a B/B_0 of 0.97 concerns the baseline concentrations when males had not yet been exposed to the females. These small errors have no impact on the final conclusions (it makes no difference if these low to undetectable values are set at 0, 0.01 or 0.02 pg), nor on the shape of the curves that are presented. The manufacturer of the RIA kit tested cross-reactivity with many steroids (androgens and other estrogens) and reported minimal or no cross-reactivity with the antibody used in this kit. The highest cross-reactivity (2.40%) was found with estrone.

Samples from different experiments were assayed in different runs with internal controls. The mean intra- and inter-assay coefficients of variation were respectively 4.36% and 9.17%.

The absolute baseline concentrations of estrogens measured across experiments 2 to 6 ranged between 0.026 and 1.463 pg/10µl. These values were however quite variable between subjects presumably based on the probe location, although true biological differences including difference in aromatase expression or response to the test environment cannot be excluded. This variability did not match directly to the location of the probe in nuclei expressing or not aromatase (H=2,561, p = 0.4644; Figure SI1). Additionally, we did not find any correlation between the estrogen content measured in baseline and the amplitude of the change detected following testosterone retrodialysis (r = 0.100, p = 0.9500, n=5), sexual interactions (POM, r = -0.032, p = 0.8743, n=26) or visual interaction with a female (BST, r = 0.008, p = 0.9907, n=9). To facilitate graphic presentation and statistical analyses, all data were therefore expressed as percentage of the concentrations measured in the last 2 or 3 time points of the baseline (BL), as it is commonly done for microdialysis studies (Hull *et al.*, 1995; Remage-Healey *et al.*, 2008; de Bournonville *et al.*, 2017).

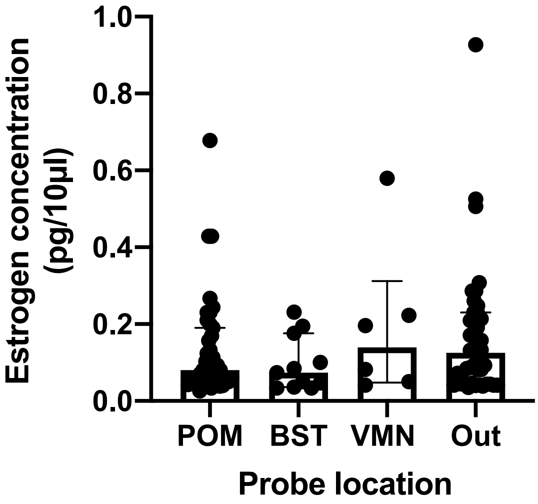

Figure SI 1 Regional variations in the concentration in estrogens in the dialysate during baseline (Mean ± interquartile ranges). POM, n = 39; BST, n = 11; VMN n = 6; Out, n = 39.

In conclusion, estrogen content measured in the dialysate in baseline conditions is somewhat variable between individuals, but as illustrated in figure SI1 the average individual values fall within a relatively narrow range in most subjects, with the exception of a few males presenting higher values. Importantly, these variations in baseline estrogen content do not predict the amplitude of the response to the treatment or the sexual stimulus. The source of individual variation in the response to the stimuli is derived from elsewhere, most probably in our opinion from a combination between individual variation in the probe location and individual variation in the response of males to the testing environment/conditions and to the sexual stimuli. Indeed, previous work showed that aromatase activity and local estrogen content is modulated by stress (Dickens *et al.*, 2011; Dickens *et al.*, 2014) and all males may not respond equally to the novel aspects of the testing environment/conditions. Moreover, not all females are equally attractive to all males and attraction or the behavioral responses it triggers are complex processes

involving several sensory modalities and neural responses that could all contribute differentially to the changes in estrogen content measured here. More work will thus be needed to understand how cues from the females or copulatory interactions with them result in changes in local estrogen concentration.

Statistical analyses

Except for experiment 1 where real concentrations were used, all data were expressed as the percentage of the last 2 or 3 points of the baseline for each subject to accommodate individual differences in absolute concentration. When one sample of the baseline of a given bird was more than 2 standard deviations away from the mean of the baseline of this bird, this sample was considered as a statistical outlier and removed from the analysis (21 points were removed on a total of 572 points for all experiments). Moreover, one sample of the post-experimental period of experiment 5 that was obviously an outlier (2310.04% of the baseline) was also removed from the analysis. For within subject analyses, removed values were replaced by the mean of their group. One sample of the interaction period in experiment 4 was lost. When time points of an individual were removed or lost, to avoid removing the whole animal in the repeated measure analyses, the removed time points were replaced by the mean value of the group at this time point.

All statistical analyses were performed by non-parametric methods using GraphPad Prism 8 due to non-normal distributions and/or significant differences in variance between groups. Friedman analyses of variance (ANOVA) for repeated measures were used to analyze changes in time of estrogen concentrations across experimental conditions in each group separately. These analyses were followed by Dunn's post-hoc tests when appropriate. To limit the number of comparisons, the last time point of the experimental period was similarly compared to the last time point of the baseline and of the post-experimental period for all experiments except for experiment 1.

Supplementary information SI2 : Calibration of dialysis probes

<u>Methods</u>

A set of *in vitro* experiments was first conducted to determine the properties of the probes and their ability to recover E_2 from baths containing known concentrations of the steroid. Dialysis probes were successively placed in baths of aCSF (without BSA) containing 0, 0.05, 0.25 or 1 ng/ml of E_2 and samples were collected every 30 min, while aCSF was perfused through the probe at a flow rate of 1 µl/min. First, probes were placed in the solution containing 0 ng/ml for 1h. Probes were then tested in ascending (starting with 0.05 ng/ml; n=3 probes) or descending (starting with 1 ng/ml; n=3 probes) concentrations of E_2 (1 h of sampling per concentration). At the end of the dialysis, probes were placed again in a solution of 0 ng/ml of E_2 for 1 h. Separate correlations were computed between E_2 concentration in the dialysate and in baths for both ascending and descending probes.

We then tested the time-course of exchanges in concentration asking when E_2 concentrations in the dialysate reach a plateau. Probes were first placed in a solution containing 0 ng/ml of E_2 for 1 h, before being placed in a solution containing 0.25 ng/ml of E_2 (n=4 probes) for 3 h. Samples were collected every 30 min again at a flow of 1 µL/min. Probes were then returned to the control solution (0 ng/ml of E_2) for 2 h. Data were analyzed by a Friedman analysis of variance followed by Dunn's post-hoc tests.

Results

A positive correlation was observed for ascending concentrations ($r=0.794$, $p=0.002$ for samples collected after 30 min of dialysis; $r=0.861$, $p<0.001$ for samples collected after 1 hour of dialysis; Figure SI 2A) and for samples collected after 1 hour of dialysis in descending concentrations ($r=0.603$, $p=0.038$; Figure SI 2B), but not for samples collected after 30 min of dialysis in descending concentrations ($r=0.292$, $p=0.358$; Figure SI 2B).

In the time-course experiment, E_2 concentration in the dialysate already increased very markedly at 30 min and began to level off after approximately 1 h even if marginal increases still occurred up to 3 h after placement in the bath containing E_2 (Figure SI 2C). The Friedman ANOVA identified a significant effect of time on the concentrations in the dialysate ($\chi^2_{n=4}=36.81$, $p<0.001$).

Conclusions

These *in vitro* validations demonstrate that expected changes in E_2 concentration can be detected by microdialysis. An increase in E_2 concentration is easily identified within 30 min, but a decrease will be detected with a longer time lag. If this result obtained *in vitro* translates to the *in vivo* situation, then the decreases in E_2 concentrations after a sexual interaction might be dampened and not reflect the true time course that would in reality be faster than detected. This means that the real effects are potentially even more dramatic than described here in our text that might minimize the dynamic nature of the responses. In the time-course experiment most of the increase in concentration was already observed at the 30 min time point. Based on these results, the *in vivo* experiments were performed with aCSF perfused at a flow rate of 1 µl/min. Samples were collected every 30 min in experiments 1-3, but every 10 min for experiments 4-6, to test whether a change in E_2 concentration could be detected faster.

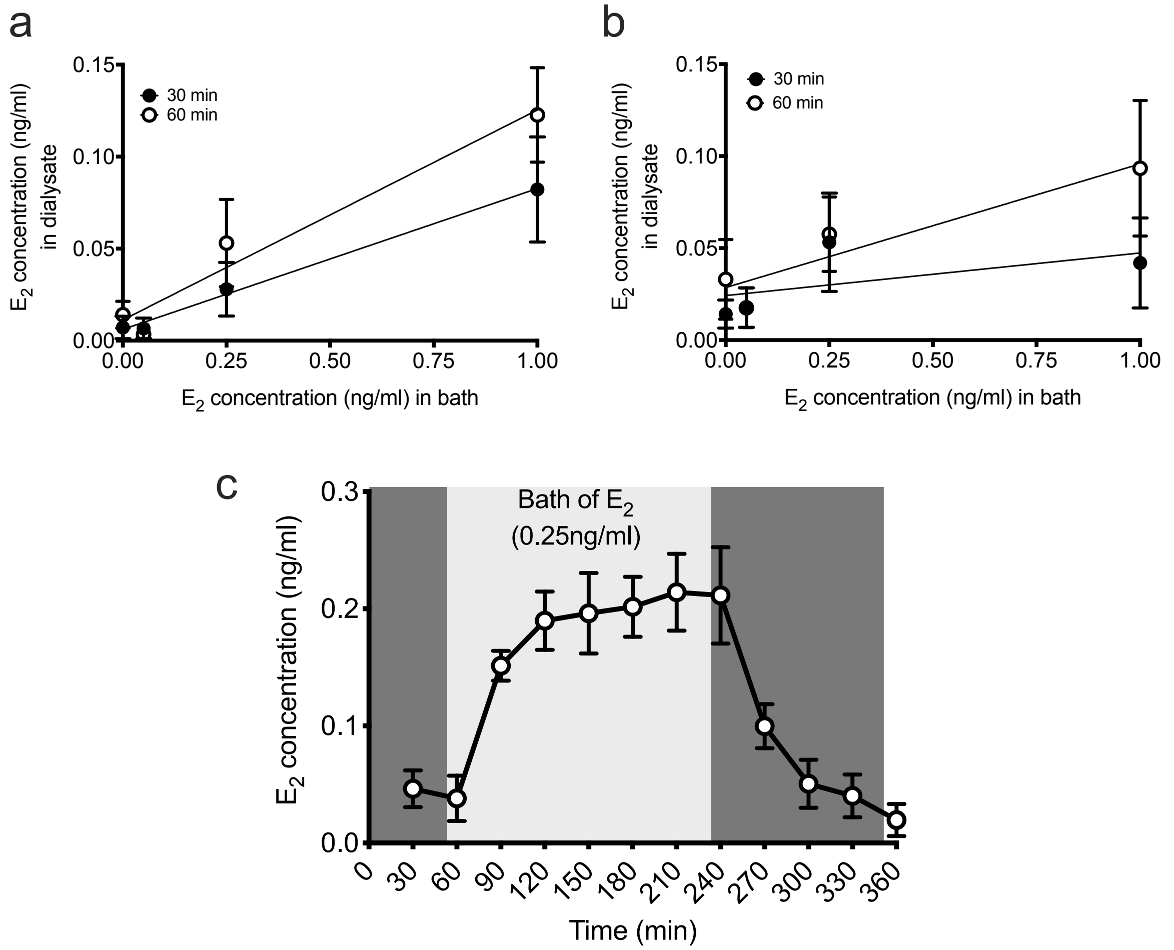

Figure SI 2: In vitro validation of the microdialysis parameters. Correlations between the concentration of E_2 measured in dialysate and the different concentrations in the bath in which the probe was immersed in ascending (A) or descending order (B). C: Changes in time of E_2 concentrations measured in the dialysate after the probe was placed in a solution at 0.25 ng/ml.

Supplementary information SI3 : Estrogen assays by ultra high performance liquid chromatography

We assayed a number of dialysates collected during experiment 3 by ultra-high performance liquid chromatography coupled with electrospray ionization tandem mass spectrometry (UHPLC-ESI-MS/MS, in short UHPLC-MS).

Methods

Samples collection

As described in the methods and results, 22 males were perfused overnight with aCSF and samples were collected at 1 µl/min every 30 min during 1 h, while the male was left undisturbed (BL), then 30 min later when the male had been allowed to interact physically with a female and finally during the next 2 h after the female had been removed from the arena.

Samples of dialysate were split in two fractions of 15 µl before being frozen at -80°C. These two fractions were then assayed either by RIA as described in the method section of the paper (see results of Experiment 3 in the paper) or by UHPLC. Multiple estrogens could be quantified by the latter method (estradiol, E_2, but also estrone, E_1, estriol, E_3 and estetrol, E_4), whereas RIA in theory quantified E_2 only, even if cross-reactions with other estrogens could take place. The technical specification coming from the company producing the assay kits indicated however limited cross-reactions with the most commonly encountered estrogens (E_1 : 2.40% ; E_3 : 0.64% ; 17α-E_2 : 0.21%).

UHPLC was used to assay the last samples of the baseline, the samples collected at the end of the interactions with the females and the first and last samples collected during the post-experimental period.

Assay technique

Chemical and reagents: Estrone (E_1), estradiol (E_2), estriol (E_3), estetrol (E_4), Bovine serum albumin (BSA), phosphate buffer saline (PBS), methyl tert-butyl ether (MTBE) and LC-MS grade ammonium fluoride (NH_4F) were purchased from Sigma-Aldrich (St-Louis, MI, USA). Deuterated E_4 (E_4-d4) and deuterated E_2 (E_2-d5) were obtained from Toronto Research Chemicals (Toronto, Canada). Acetonitrile (ACN), methanol (MeOH), water (H_2O) of ULC-MS grade and LC-MS grade isopropanol (IPA) were purchased from Biosolve (Valkenswaard, the Netherlands). E_1, E_2, E_3, E_4, E_4-d4 and E_2-d5 were individually weighed using a XPR microbalance (Mettler Toledo, Columbus, OH, USA) and dissolved in MeOH at a concentration of 0.1 mg/ml. They were subsequently diluted with MeOH to 10 µg/ml, aliquoted and stored at -80°C.

Calibration curves: Stock of E_1, E_2, E_3 and E_4 were mixed and diluted to 1 µg/ml using a mixture of H_2O/MeOH (75/25 v/v). Then, the solution was diluted to 100 ng/ml using a solution of 10% BSA in PBS. This solution was subsequently diluted to make 8 calibration levels (from 5 pg/ml to 100 ng/ml). Calibration curves underwent the same extraction process as samples.

Sample preparation: Samples were defrosted at room temperature and transferred in 1.5 ml microtubes. Then, 15 µl of internal standard (50 pg/ml) and 30 µl of H_2O were added and vortex-mixed. 50 µl of MTBE was added and the solutions were vortex-mixed for 15 min. The samples were centrifuged at 13 000 RPM for 5 min then placed at -80°C for 15 min to freeze the aqueous layer. The organic layer was subsequently collected in 1.5 ml centrifuge tubes. The extraction was repeated twice and the organic layers were pooled and evaporated to dryness using a vacuum concentrator at 40°C (LabConco, Kansas-City, MO, USA). The samples were reconstituted with 20 µl of a mixture of H_2O/MeOH (75/25 v/v).

Chromatographic conditions: The UHPLC technique used a 1290 Infinity LC system coupled to a 6495 triple quadripole mass spectrometer equipped with the iFunnel technique (Agilent technologies). The chromatographic separation was performed on a reverse phase Zobrax C18 column (1.8 µm, 50x2.1 mm ID; Agilent Technologies) thermostated at 60°C in gradient mode with two mobile phases A (0.1 mM ammonium fluoride of LC-MS [NH4F] quality, Sigma Aldrich in H_2O) and B (MeOH/Acetonitrile [ACN], Biosolve, 1:1) flowing at 0.25 ml/min. The gradient started with 20% phase B, increased to 90% phase B between 0 and 4 min, stayed at these values between 4 and 6 min and finally decreased back to 20 % phase B between 6

and 6.1 min. The column was then equilibrated at 20% phase B for 3 min before the next sample was injected. To limit cross contaminations between samples, the injection needle was cleaned (inside and outside) with 10 µl of a mixture of H_2O/MeOH/ACN/Isopropanol (1/1/1/1 v/v) LC-MS quality (IPA) between each injection. The outside of the needle was also cleaned with a solution of H2O/MeOH/isopropanol (2/4/4 v/v). The autosampler was thermostated at 6°C and kept in the dark). 5 µl of each sample was injected in duplicate for each sample.

Mass spectrometry: The electrospray was operated in negative ionization mode (ESI) and conditions were optimized with the Source Optimizer software included in the MassHunter software (Agilent Technologies). The capillary and nozzle voltage were set at -2000 V. Nitrogen was used as dry gas heated at 200°C with a flow rate of 11l/min and at 400°C at 12 l/min respectively. The nebulizer pressure was set at 55 psi. The high and low pressure funnels were operated at 190 and 120 V. Fragmentation and collision energies were optimized for each analyte with the Optimizer software. Unit mass resolution was set in both mass-resolving quadrupoles Q1 and Q3. Cell accelerator voltage was set at 3 V. Analyses were carried out in dynamic multiple reactions monitoring (dMRM) mode. Results were acquired with the MassHunter Data Acquisition software and subsequently analyzed with the Quantitative Analysis.

Statistical analyses

Data were analyzed with the GraphPad Prism 8 software. Correlations were computed between concentrations of E_1, E_2 or E_1+E_2 in UHPLC results and concentrations of E_2 measured in RIA. Some samples (17 out of 88) had however to be removed from the analysis because E_2 in RIA was below the detection limit (they were set at the detection limit in the presentation of results of Experiment 3) or because they were more than 2 standard deviations away from other results.

Results

Initially UHPLC-MS was programmed to only detect E_1 and E_2 in the samples. The concentration of E_1 or of E_1+E_2 in UHPLC assays did not correlate with the E_2 measured in RIA (r=-0.22, p=0.0906, r=-0.15, p=0.2360, respectively). In contrast, E_2 concentrations measured by the two methods were positively correlated (r=0.46, p=0.0002), but the slope of the regression between these measures was lower than 1 (y=0.2112 X + 0.1578), indicating that UHPLC-MS systematically detected lower concentrations than RIA (see Figure SI 3).

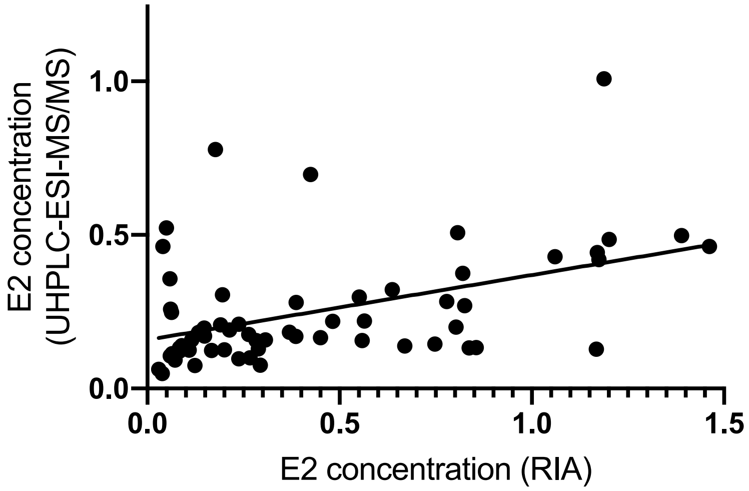

Figure SI 3: Correlation between the concentrations (pg/10 µl) of E_2 measured by the two assay methods RIA and UHPLC

Additional analyses were then carried out to identify the different estrogens and their metabolites present in the dialysates (see Figure SI 4 for a representative chromatogram). To this end, the technique was modified to include a derivatization step with dansyl chloride to enhance sensitivity. Briefly, 10 µL samples were added to 20 µL internal standard (E_2-d5 and E_4-d4) and subsequently were extracted with 2 times 50 µL ethyl acetate. The two organic fractions were pooled and evaporated to dryness (65°C, 15 min) in a vacuum concentrator. Then, 10 µl Na_2CO_3 0.1 M and 10 µL dansyl chloride (1 mg/mL in ACN) were added to the tubes and incubated for 30 min at 65°C. The derivatization was quenched with 5 µL formic acid before evaporating to dryness (65°C, 15 min) in a vacuum concentrator. Finally, the derivatized samples were reconstituted in 20 µl H_2O/ACN/TFA (75:25:1). Calibrant solutions containing 2-OH E_1, 4-OH E_1, 2-OH E_2, 4-OH E_2, 6-OH E_2, 2-OH E_3, E_1, E_2, E_3 and E_4 in aCSF were prepared at concentrations at 200, 100, 50, 20, 10, 5, 2, 1 pg/mL.

Samples were analyzed by UHPLC-MS with mobile phases composed of 0.1% formic acid (A) and ACN + 0.1% FA (B) on a F5 (2.1 x 100 mm, 1.7 µm particle size) (Phenomenex, Torrance, CA, USA) at a flow rate of 0.4 mL/min. Column was thermostated at 55°C. Gradient ramped from 45% B to 70% B over 7 min. 20 µL of the samples were injected.

The transitions that were followed are described in table SI1.

Table SI1 : Transitions followed used for the identification and quantitation of estrogenic metabolites by UHPLC-MS.

**Compound**	**Precursor ion m/z**	**Product ion m/z**
E4	538.4	171
E3	522.4	171, 458
17-β E2	506.4	171, 491
17-α E2	506.4	171
E1	504.4	171, 425
2-OH E1	520.4	171, 170, 519
4-OH E1	520.4	171
16-α OH E1	520.2	171, 505
2-Me E1	534.4	171

4-Me E1	534.4	171, 470
2-OH E2	522.4	171, 170, 521
4-OH E2	522.4	171
6-OH E2	522.4	171
2- Me E2	536.4	171, 472
2-OH E3	538.4	171
17-α epi E3	522.4	171
16-α epi E3	522.4	171
16-β 17-α epi E3	522.4	171

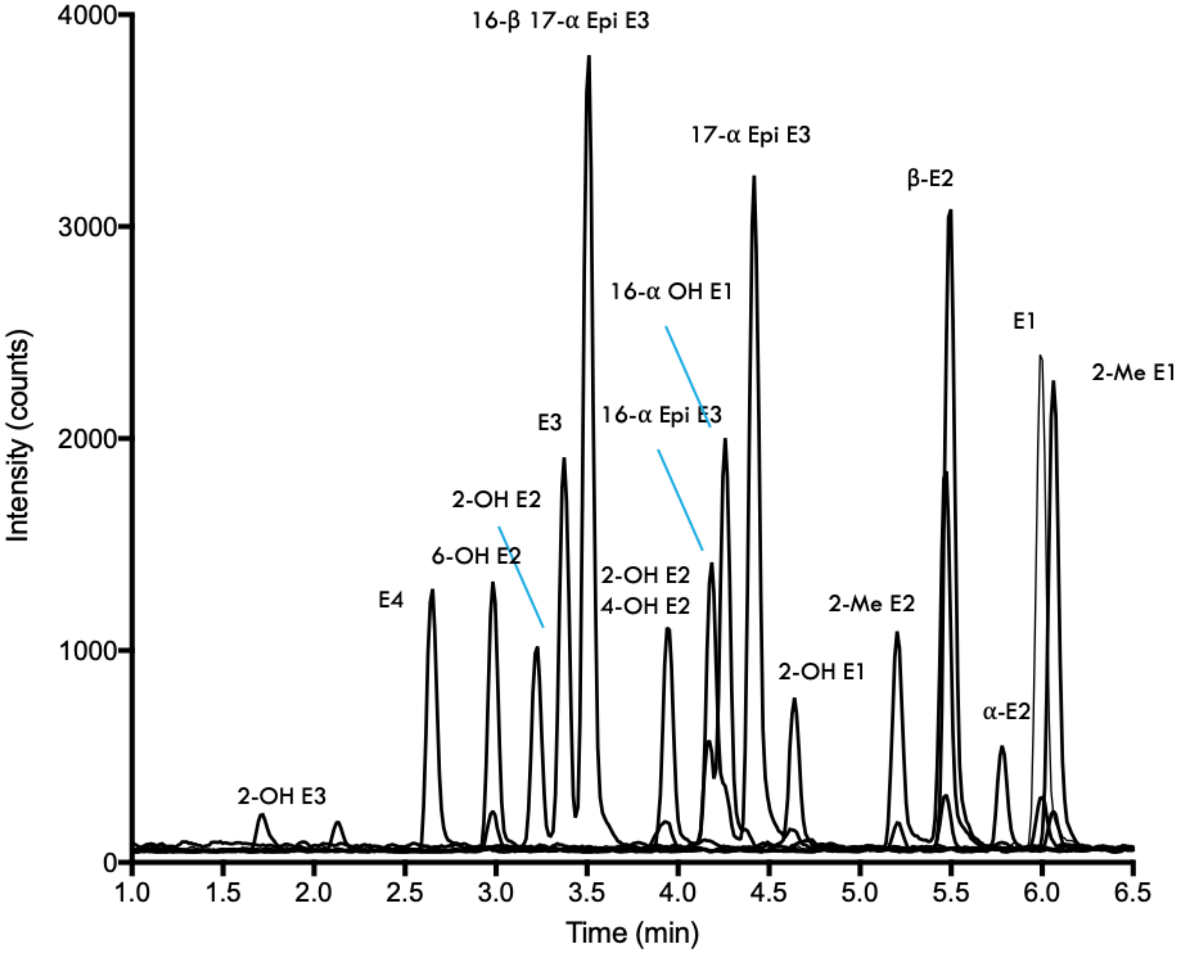

Figure SI 4: Example of chromatogram illustrating our ability to identify a mix estrogens present at 100µg/ml in a clean medium by UHPLC-MS each after derivatization with dansyl chloride. Estrone (E_1), Beta-estradiol (17β-E_2), alpha-estradiol (17α-E_2), Estriol (E_3), estetrol (E_4), 2-hydroxy estrone (2-OH E_1), 16-hydroxy alpha estrone (16-α OH E_1), 2-methyl estrone (2-ME E_1), 2-hydroxy estradiol (2-OH E_2), 4-hydroxy estradiol (4-OH E_2), 6-hydroxy estradiol (6-OH E_2), 17-alpha epi estriol (17-α epi E_3), 16-alpha epi estriol (16-α epi E_3), 16-beta epi estriol (16-β-epi E_3). 2-OH E2 generates two peaks as two phenol hydroxyls are available for dansylation.

The most concentrated estrogens that were detected are reported in Table SI2. These analyses interestingly indicate that other estrogens besides E_2 are present in

much higher amounts in the dialysates. E_2 only represents a small fraction of the total (19.6%) of the 5 steroids listed in the table but would even be a lower fraction if all estrogens present in the chromatogram were included in the sum.

Estrogens	Concentration (pg/ml)
E1	0.33 ± 0.15
E2	0.95 ± 0.56
E3	0.31 ± 0.15
2-OH-E1	2.54 ± 0.48
2-OH-E2	N.D.
16-17🚲-✓EPI E3	0.71 ± 0.3

Table SI 2: Concentrations of the most abundant estrogens in dialysates collected in baseline conditions (n=3). Data are represented as means ± SEM. N.D.= not detected

Discussion

These assays identified a positive correlation between concentrations of E_2 measured by RIA and UHPLC. However, their critical analysis strongly suggests that the two methods do not measure exactly the same thing since values obtained by UHPLC are systematically smaller than those measured by RIA. The reasons explaining these discrepancies remain unclear at present despite multiple attempts to resolve the question.

Since the RIAs systematically detected higher concentrations of E_2 and these RIAs were performed on non-extracted samples, one could imagine that one unidentified compound present in the dialysates systematically blocked the binding of the iodinated E_2 to the antibody which is interpreted as additional E_2 when reading the results of the standard curve. A number of assays in which we compared E_2 concentrations measured by RIA in samples that were not extracted or were extracted by the method used for UHPLC-MS did not however detect major differences that could explain this phenomenon: if anything values after extraction (0.49 ± 0.10 pg/10µl) were slightly higher (not lower) than without extraction (0.38 ± 0.40 pg/10µl). Therefore, the most likely explanation for the higher concentrations measured in UHPLC-MS compared to RIA is that due to its lower specificity with regard to the estrogen species RIA provides a more global measure of estrogens (Jalabert *et al.*, 2020).

It is important to recall that the E_2 peaks observed after copulation or visual interaction with a female display an anatomical- (only observed for specific probe locations), temporal- (only observed during the period of interaction and immediately after) and stimulus- (different response to the view or physical interaction with the female) specificity. Furthermore, the *in vitro* and *in vivo* validations clearly indicated that the RIA of dialysates accurately detects changes in E_2 concentrations in the incubation medium *(in vitro*), detects a systemic injection of E_2 in a live animal and detects an increased E_2 brain concentration after retrodialysis of testosterone ONLY if the probe is located in a brain expressing aromatase. These multiple validations strongly support the idea that the peaks observed in POM after copulation or in BST after visual exposure to a female represent *in vivo* changes in estrogen concentrations. It remains possible however that these assays did not only measure E_2. The antibody used in these assays is highly specific according to the

manufacturer and only presents minimal cross-reactions with other estrogens (Estrone: 2.40%, Estrone-β-D-glucuronide: 0.20%, Estrone-3-sulfate: 0.01%, Equilin: 0.34%, D-Equilenin: 3.40%, 17α-estradiol: 0.21%, 17β-estradiol-3-glucuronide: 2.56%, estradiol-3-SO_4: 0.17%, Estriol: 0.64%). It remains however possible that another estrogen, not tested in these specificity assays, or another compound of low molecular weight (below 6kDa, the cut-off of the dialysis probe) is detected by the antibody. If that is the case, it is then likely that this compound is an estrogen produced by local aromatization, very probably a metabolite of E_2, since the peaks were only observed in aromatase-rich brain areas. Given the extremely low concentrations that were measured in these experiments (always below 1 pg/tube) the identification of such compounds is presently very difficult, if not impossible.

References

Bardet, S.M., Cornil, C.A. & Balthazart, J. (2010) Testosterone recruits new aromatase-imunoreactive cells in neonatal quail brain. *NeuroReport*, **21**, 376-380.

Carere, C., Ball, G.F. & Balthazart, J. (2007) Sex differences in projections from preoptic area aromatase cells to the periaqueductal gray in Japanese quail. *Journal of Comparative Neurology*, **500**, 894-907.

de Bournonville, C., Smolders, I., Van Eeckhaut, A., Ball, G.F., Balthazart, J. & Cornil, C.A. (2017) Glutamate released in the preoptic area during sexual behavior controls local estrogen synthesis in male quail. *Psychoneuroendocrinology*, **79**, 49-58.

Dickens, M.J., Cornil, C.A. & Balthazart, J. (2011) Acute stress differentially affects aromatase activity in specific brain nuclei of adult male and female quail. *Endocrinology*, **152**, 4242-4251.

Dickens, M.J., de Bournonville, C., Balthazart, J. & Cornil, C.A. (2014) Relationships between rapid changes in local aromatase activity and estradiol concentrations in male and female quail brain. *Hormones and behavior*, **65**, 154-164.

Foidart, A., Reid, J., Absil, P., Yoshimura, N., Harada, N. & Balthazart, J. (1995) Critical re-examination of the distribution of aromatase-immunoreactive cells in the quail forebrain using antibodies raised against human placental aromatase and against the recombinant quail, mouse or human enzyme. *J.Chem.Neuroanat.*, **8**, 267-282.

Hull, E.M., Du, J., Lorrain, D.S. & Matuszewich, L. (1995) Extracellular dopamine in the medial preoptic area: implications for sexual motivation and hormonal control of copulation. *Journal of Neuroscience*, **15**, 7465-7471.

Jalabert, C., Ma, C. & Soma, K.K. (2020) Profiling of systemic and brain steroids in male songbirds: Seasonal changes in neurosteroids. *Journal of neuroendocrinology*, e12922.

Newman, S.W. (1999) The medial extended amygdala in male reproductive behavior - A node in the mammalian social behavior network. *Ann.NY Acad.Sci.*, **877**, 242-257.

Remage-Healey, L., Maidment, N.T. & Schlinger, B.A. (2008) Forebrain steroid levels fluctuate rapidly during social interactions. *Nat Neurosci*, **11**, 1327-1334.
